# Nanoblock-mediated selective oncolytic polypeptide therapy for triple-negative breast cancer

**DOI:** 10.7150/thno.81834

**Published:** 2023-05-08

**Authors:** Cuiyu Zhong, Jie Li, Suiping Liu, Weirong Li, Qiang Zhang, Junpeng Zhao, Menghua Xiong, Yan Bao, Yandan Yao

**Affiliations:** 1 Breast Tumor Center, Sun Yat-sen Memorial Hospital, Sun Yat-sen University, Guangzhou 510120, China.; 2 Guangdong Provincial Key Laboratory of Malignant Tumor Epigenetics and Gene Regulation, Guangdong-Hong Kong Joint Laboratory for RNA Medicine, Medical Research Center, Sun Yat-Sen Memorial Hospital, Sun Yat-Sen University, Guangzhou 510120, China.; 3 Shenshan Medical Center, Sun Yat-sen Memorial Hospital, Sun Yat-sen University, Shanwei 516621, China.; 4 Nanhai Translational Innovation Center of Precision Immunology, Sun Yat-Sen Memorial Hospital, Foshan 528200, China.; 5 School of Biomedical Sciences and Engineering, South China University of Technology, Guangzhou International Campus, Guangzhou, 511442, China.; 6 The Third Affiliated Hospital, Guangzhou Medical University, Guangzhou 510150, China.; 7 Faculty of Materials Science and Engineering, South China University of Technology, Guangzhou, 510640, China.

**Keywords:** Oncolytic polypeptides, Triple-negative breast cancer, Tumor microenvironment, pH responsiveness

## Abstract

**Rationale:** Broad-spectrum oncolytic peptides (Olps) constitute potential therapeutic options for treating heterogeneous triple-negative breast cancer (TNBC); however, their clinical application is limited owing to high toxicity.

**Methods:** A nanoblock-mediated strategy was developed to induce selective anticancer activity of synthetic Olps. A synthetic Olp, C12-PButLG-CA, was conjugated to the hydrophobic or hydrophilic terminal of a poly(ethylene oxide)-*b*-poly(propylene oxide) nanoparticle or a hydrophilic poly(ethylene oxide) polymer. A nanoblocker, that can significantly reduce the toxicity of Olp, was screened out through hemolytic assay, and then Olps were conjugated to the nanoblock via a tumor acidity-cleavable bond to obtain the selective ^R^Nolp ((mPEO-PPO-CDM)_2_-Olp). The tumor acidity responsive membranolytic activity, *in vivo* toxicity and anti-tumor efficacy of ^R^Nolp were determined.

**Results:** We found that the conjugation of Olps to the hydrophobic core of a nanoparticle but not the hydrophilic terminal or a hydrophilic polymer restricts their motion and drastically reduces their hemolytic activity. We then covalently conjugated Olps to such a nanoblock via a cleavable bond that can be hydrolyzed in the acidic tumor environment, yielding a selective ^R^Nolp molecule. At physiological pH (pH 7.4), ^R^Nolp remained stable with the Olps shielded by nanoblocks and exhibited low membranolytic activity. At the acidic tumor environment (pH 6.8), Olps could be released from the nanoparticles via the hydrolysis of the tumor acidity-cleavable bonds and exerted membranolytic activity against TNBC cells. ^R^Nolp is well tolerated in mice and demonstrated high antitumor efficacy in orthotopic and metastatic mouse models of TNBC.

**Conclusion:** We developed a simple nanoblock-mediated strategy to induce a selective cancer therapy of Olps for TNBC.

## Introduction

Triple-negative breast cancer (TNBC) is a breast cancer subtype with the least favorable prognosis, largely owing to the paucity of effective therapies besides chemotherapy 1-5. The remarkable heterogeneity of genomic, transcriptomic, and proteomic characteristics and stromal cell composition of this subtype make broad-spectrum options particularly attractive for TNBC therapy 1, 6-13.

Oncolytic peptides (Olps), a type of traditional cationic, amphiphilic molecules, provide potential anti-cancer therapeutic options with broad-spectrum 14-21. Their cationic residues offer strong binding capacity with negatively charged cell membrane surfaces and encourage hydrophobic fragment insertion into and disruption of cell membranes, causing intracellular content leakage and cell death 22. Olps tend to target cancer cells because of the display of anionic phosphatidylserine on their outer cell membrane 14. These peptides exhibit potent cytotoxicity against various cancer cells regardless of heterogeneity or drug resistance 21, 22. In addition, the membrane disruption mechanism of Olps decreases the possibility of developing drug resistance compared with traditional antineoplastic agents 21, 23, 24. Thus, Olps shows notable potential for effective cancer treatment 15, 23, 25, 26. However, Olps also show high toxicity to normal cells, and the off-target toxicity limits their clinical application only to intratumor injections 20, 27.

The membranolytic blocks (MBs) containing cationic and amphiphilic moieties are not only the key to the antineoplastic activity of Olps, but also underlie their toxic side effects on normal tissues 28. To increase the selectivity of Olps towards tumor tissues and decrease their toxicity to normal tissues, selective Olps were developed through responsive modulation of MBs in respect of their cationicity, hydrophobicity, amphipathicity and structural propensity in tumor and normal tissues 18. Human cathelicidin peptide LL-37 is stored in the human body as a precursor protein hCAP18 that induces steric hindrance toward the direct contact between LL-37 and cell membranes 29. Active LL-37 peptide is released via specific protease activities, subsequently disrupting target cell membranes 30, 31. Here, we investigated whether and how a simple spatial shield of synthetic Olps could prevent their interaction to cell membranes and reduce their toxicity in normal tissues. And if such a shield were labile in the tumor microenvironment, cancer cells could be selectively exposed to Olps and thus be killed.

**Figure 1:**
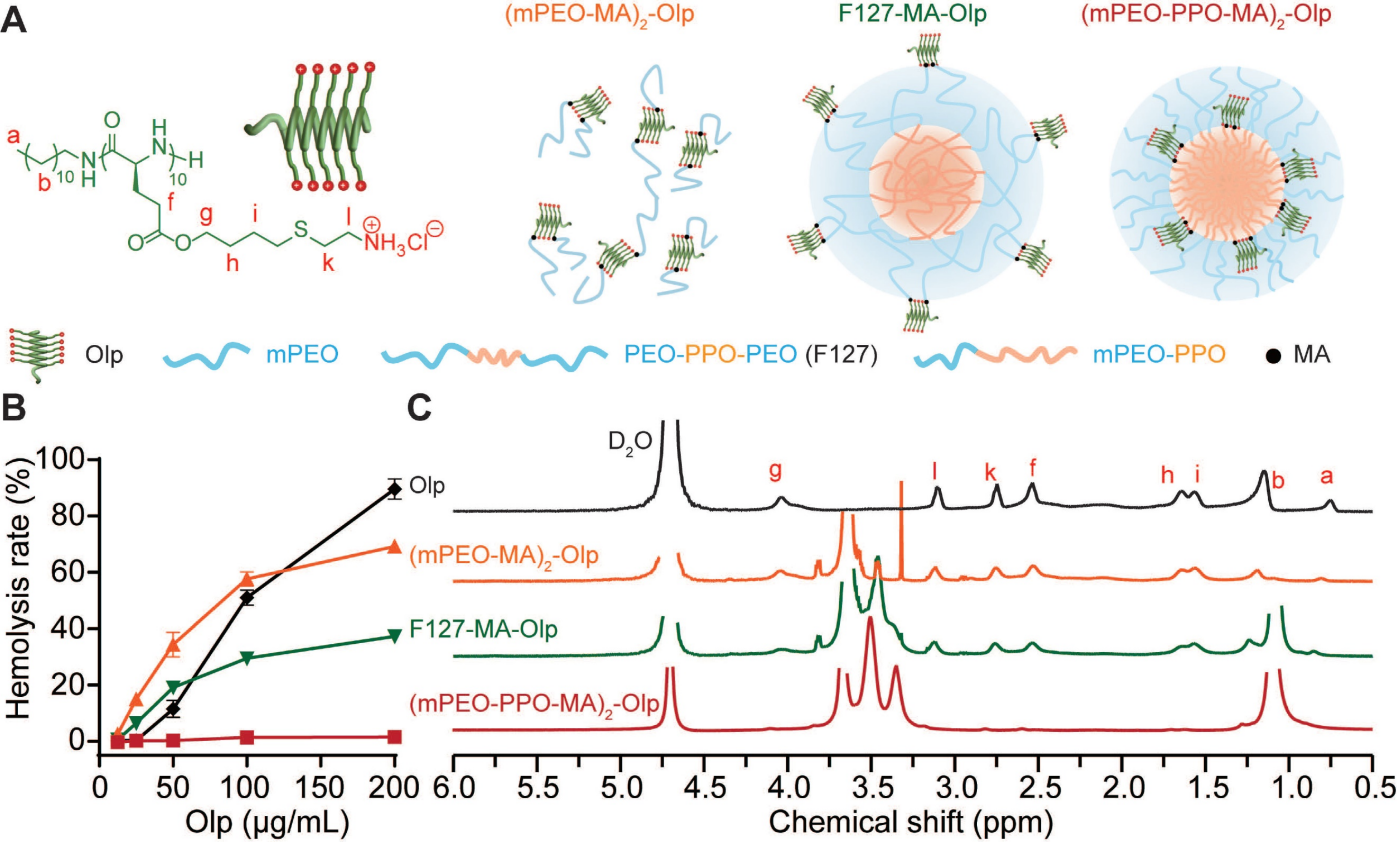
** Nanoblock-mediated shielding of Olp. A**, Chemical structure of the synthesized Olp and schematic diagram of (mPEO-MA)_2_-Olp, F127-MA-Olp, and (mPEO-PPO-MA)_2_-Olp. **B**, Concentration-dependent hemolytic activity of Olp, (mPEO-MA)_2_-Olp, F127-MA-Olp, and (mPEO-PPO-MA)_2_-Olp after 1 h incubation at 37 °C. **C**, ^1^H NMR spectra of Olp, (mPEO-MA)_2_-Olp, F127-MA-Olp, and (mPEO-PPO-MA)_2_-Olp in D_2_O. In **B**, data are presented as the mean ± s.d. (n = 3).

We report a simple biomimetic strategy to induce selective anticancer activity of synthetic Olps via the spatial shielding of Olps using a nanoblock and incorporating a linker to allow the release of Olps in the acidic tumor environment 32. We found that the conjugation of Olps to the hydrophobic terminal of an amphiphilic block polymer of poly(ethylene oxide)-poly(propylene oxide) (mPEO-PPO) considerably reduced the hemolytic activity of Olps (**Figure 1A**). Then, the selective ^R^Nolp was subsequently constructed by conjugating Olps to the PPO terminal of mPEO-PPO via carboxy-dimethylmaleic anhydride (CDM) that could be cleaved in tumor acidity. At pH 7.4, ^R^Nolp assembled into nanoparticles with Olps shielded by PEO shells and exhibited minimal hemolytic activity. At pH 6.8, Olps were released following the cleavage of the CDM linker, exhibiting potent membranolytic activity and cytotoxicity. Last, we verified that the intravenous administration of ^R^Nolp inhibited tumor growth in orthotopic and metastatic mouse models of TNBC. Our study provides a nanoblock-mediated strategy to enhance the selectivity of Olps for anti-TNBC therapy.

## Methods

### Synthesis of dodecyl-poly(γ-(3-butenyl alcohol)-L-glutamate) (C12-PButLG)

But-L-Glu-based N-carboxyanhydride (But-L-Glu-NCA) was synthesized according to a published procedure 27. But-L-Glu NCA (1.0 g, 4.4 mmol) was dissolved in dimethylformamide (DMF) (10.0 mL). Then, dodecylamine (77.0 mg, 0.415 mmol) was added to initiate the reaction in a glovebox. After stirring at room temperature for 28 h, C12-PButLG was obtained via precipitation in cold diethyl ether and hexane (1:1, v/v). The precipitate was dried under vacuum overnight (588.0 mg, yield: 70.2%). The *M_n_* (2017 g/mol) of C12-PButLG was determined by ^1^H NMR.

### Synthesis of C12-PButLG-CA (Olp)

C12-PButLG (200.0 mg, 0.98 mmol of alkene groups), cysteamine hydrochloride (337.8 mg, 2.96 mmol), and 2,2-dimethoxy-2-phenylacetophenone (12.0 mg, 0.048 mmol) were dissolved in DMF (3.0 mL). N_2_ was used to purge the solution for 30 min, followed by UV irradiation (λ_max_ = 365 nm) for 60 min. The product was obtained via dialysis (MWCO = 1 kDa) against distilled water for 24 h followed by lyophilization (216.0 mg, yield: 78.3%).

### Synthesis of mPEO-PPO-CDM

A block copolymer of mPEO-PPO was synthesized according to a published procedure 33. 2-Propionic-3-methylmaleic anhydride (carboxy dimethylmaleic anhydride, CDM) was synthesized as previously described 34. CDM (20 mg, 0.11 mmol) was dissolved in dichloromethane (4 mL). The solution was mixed with oxalyl chloride (18 mg, 1.35 mmol) with a catalytic amount of DMF (25 µL). Then, the solution was stirred for 10 min in an ice-water bath followed by 2 h at room temperature. To obtain 3-Furanpropanoyl chloride (CDM-Cl), the solution was vacuum-dried. Then, CDM-Cl was dissolved in dichloromethane (4 mL) and mPEO-PPO (1.0 g, 0.057 mmol) was added. The mixture was stirred for 10 min in an ice-water bath and then for 2 h at room temperature. The reaction was quenched with a saturated ammonium chloride aqueous solution (20 mL). Then, it was extracted with CHCl_3_ (20 mL) 3 times. The organic layer was combined and concentrated to 2 mL and then precipitated in cold ethyl ether. The product was filtered and dried overnight in vacuum (yield: 70%). The *M_n_* (17200 g/mol) of mPEO-PPO-CDM was determined by ^1^H NMR.

### Synthesis of (mPEO-PPO-CDM)_2_-Olp

C12-PButLG-CA (40 mg, 0.014 mmol) was dissolved in methanol (2 mL). Then, mPEO-PPO-CDM (500 mg, 0.028 mmol) was dissolved in 10 mL CH_2_Cl_2_ and mixed with the C12-PButLG-CA solution at room temperature while stirring. After 24 h, the mixture was added to cold ethyl ether. The product was filtered and dried overnight in a vacuum (yield: 90%).

### Synthesis of (mPEO-PPO-MA)_2_-Olp, (mPEO-MA)_2_-Olp, and F127-MA-Olp

mPEO-PPO-MA (1.0 g, 0.056 mmol) was dissolved in 6 mL dichloromethane. Then, oxalyl chloride (100 μL) and DMF (10 μL) were added to catalyze the reaction for 30 min in an ice-water bath and then for 2 h at room temperature. DMF and excess oxalyl chloride were removed to obtain mPEO-PPO-MA-Cl. Methanol (2 mL) was used to dissolve C12-PButLG-CA (80.0 mg, 0.028 mmol). Then, mPEO-PPO-MA-Cl and triethylamine (20 μL) were added. After 24 h of reaction, the mixture was concentrated and precipitated in diethyl ether in an ice-water bath to obtain the non-responsive polypeptide (mPEO-PPO-MA)_2_-Olp. (mPEO-MA)_2_-Olp and F127-MA-Olp were synthesized using a similar method as (mPEO-PPO-MA)_2_-Olp.

### Preparation of nanoparticles

(mPEO-MA)_2_-Olp, F127-MA-Olp, (mPEO-PPO-MA)_2_-Olp (^NR^Nolp), and (mPEO-PPO-CDM)_2_-Olp (^R^Nolp) were dissolved in dimethylsulfoxide (DMSO) and added to phosphate-buffered saline (PBS) buffer (pH 7.4) dropwise while stirring (400 rpm). Then, the mixtures were dialyzed in PBS buffer (pH 7.4) at room temperature for 4 h to remove DMSO (MWCO = 3500 Da). The resultant mixtures were collected and diluted to the desired concentration.

### ^1^H NMR detection of Olp conjugates

(mPEO-MA)_2_-Olp, F127-MA-Olp, ^NR^Nolp, and ^R^Nolp were dissolved in DMSO-d_6_ and added to D_2_O dropwise while stirring (400 rpm). By refilling with D_2_O, the solutions were purified with a Microsep (30 kDa). Then, proton nuclear magnetic resonance (^1^H NMR) spectroscopy was used to characterize the solutions.

### Nanoparticle size at different pH conditions

^R^Nolp or ^NR^Nolp particles were diluted to 156 μg/mL in PBS buffer of pH 7.4 or 6.8. The particle solutions were incubated at 4 ℃ for 2 h, and their diameters were measured.

### Animals

Institute of Cancer Research (ICR) mice (6-8 weeks) and BALB/c mice (6-8 weeks) were purchased from Hunan SJA Laboratory Animal Co., Ltd. (China). All animal experiments were performed in accordance with the guidelines outlined in the Guide for the Care and Use of Laboratory Animals. Mice were housed under specific, pathogen-free conditions. All mice were fed a standard pellet diet and water ad libitum. The procedures were approved by the South China University of Technology Animal Care and Use Committee.

### Annexin V-FITC/PI apoptosis assay

At a density of 2 × 10^5^ cells/well, 4T1 cells were seeded into 6-well plates. After overnight incubation, the cell culture medium was replaced with fresh medium containing Olp (5 μg/mL) or ^NR^Olp (5 μg/mL of Olp). After 24 h incubation, the cells were digested into single-cell suspensions with EDTA-free trypsin and then washed twice with PBS. Harvested cells were stained according to the manufacturer's instructions provided with the Annexin V-FITC/PI apoptosis detection kit. The stained cells were characterized within 10-15 min using a Cytoflex flow cytometer (Beckman Cytoflex, USA). Data were analyzed using the CytExpert software (Beckman Coulter, USA).

### Determination of maximum tolerated dose (MTD)

Female ICR mice (6 weeks old) were intravenously (i.v.) injected with various doses of ^R^Nolp or Olp (8 mice for each group) and their survival was observed for 3 days. MTD was defined as the maximum dose that does not cause drug-related death.

### Orthotopic EMT6 and 4T1 tumor treatments

For EMT6 and 4T1 breast cancer models, 2 × 10^5^ cells suspended in 50 μL PBS were injected orthotopically into the second left mammary fat pad of female BALB/c mice (6-7 weeks old). The perpendicular diameters of the tumors were measured using calipers. The tumor volume was calculated using the formula: tumor volume (mm^3^) = (length × width^2^) × 1/2. When the average tumor volume reached ~50 mm^3^, the mice were divided into groups with approximately equal mean tumor volume. For the EMT6 tumor model, the mice were divided into 3 groups (n = 7 per group) and i.v. injected with PBS, ^R^Nolp (6 mg/kg of Olp), or ^NR^Nolp (6 mg/kg of Olp) daily for 12 injections. On day 12 after the start of treatment, the mice were euthanized. For the 4T1 tumor model, the mice were divided into 3 groups (n = 6 per group) and i.v. injected with PBS, ^NR^Nolp (6 mg/kg of Olp) or ^R^Nolp (6 mg/kg of Olp) daily for 12 days. On day 13 after the start of treatment, the mice were euthanized. Then, the tumor tissues were resected, weighed, and photographed.

### Metastasis treatment

4T1 cells (5 × 10^4^ cells per mouse) were i.v. injected into BALB/c mice (female, 6-7 weeks old; Hunan SJA Laboratory Animal Co., Ltd., China). Four days later, the mice were randomly divided into 3 groups (5 independent mice per group) following i.v. injection of PBS, ^NR^Nolp (6 mg/kg of Olp) or ^R^Nolp (6 mg/kg of Olp) daily for 12 days and then every other day for 4 days. The weight of the mice was recorded every other day until they were euthanized on day 21 after tumor cell injection. The lung and liver tissues were collected and fixed with 4% paraformaldehyde (PFA). The lung tissues were photographed. Randomly selected lung and liver tissues of two mice were used for H&E staining.

### Liver and kidney toxicity test

Female ICR mice (6 weeks old) were i.v. injected with PBS, ^R^Nolp (6 mg/kg of Olp), or ^NR^Nolp (6 mg/kg of Olp) daily for 3 days. After 7 days, serum was collected for the detection of alanine aminotransferase (ALT), aspartate aminotransferase (AST), albumin (ALB), serum creatinine (CREA), and urea using an automatic biochemical analyzer (Hitachi 3100, Hitachi, Tokyo, Japan).

### Statistical analysis

Data were expressed as means ± s.d. for *in vitro* experiments and expressed as means ± s.e.m. for *in vivo* experiments. GraphPad Prism 8 software (GraphPad Software Inc) was used for statistical analysis. Unpaired two-tailed Student's t-tests was performed for analyze the differences between groups. n.s. indicating no significant difference. Asterisk (*) indicated a significant difference (* P < 0.05, ** P < 0.01, *** P < 0.001).

## Results and Discussion

### Nanoblock-mediated shielding of Olps

We synthesized a radially amphiphilic polypeptide C12-PButLG-CA with pendant amine groups through dodecylamine-initiated *N*-carboxyanhydride (NCA) polymerization and thiol-ene reaction (**Scheme S1 and Figure S1-2**) 27. The resultant C12-PButLG-CA was highly water-soluble with a stable helical structure (**Figure S3**). The cytotoxicity of this Olp was evaluated against TNBC cell lines of mouse origin (4T1 and EMT6) and human origin (MDA-MB-231) (**Figure S4A**). This Olp exhibited potent cytotoxicity against all the evaluated TNBC cell lines, with IC_50_ values (50% inhibitory concentrations) < 20 μg/mL after 1 h of treatment (**Figure S4A**). Olp-treated 4T1 cells were both positive for Annexin V and PI staining after 24 h of treatment, indicating necrotic cell death with cell membrane damage (**Figure S4B**). However, this Olp also exhibited potent hemolytic activity (**Figure 1B**). Thus, we obtained a synthetic Olp with potent cytotoxicity against TNBC cells but also high hemolytic activity.

We investigated various methods to reduce the toxicity of this Olp using simple spatial shielding. We conjugated this Olp to the hydrophilic mPEO_5k_ or to the hydrophilic or hydrophobic terminal of block polymers that self-assemble into nanoparticles. Then, we generated conjugates (mPEO-MA)_2_-Olp (**Scheme S2 and Figure S7**), F127-MA-Olp (**Scheme S3 and Figure S8**), and (mPEO-PPO-MA)_2_-Olp (**Scheme S4 and Figure S9**). Following conjugation, the Olp retained its helical structure (**Figure S10A**). The zeta potential of all the prepared nanoparticles was < 5 mV (**Figure S10B**). The conjugation of Olp to mPEO_5k_ or the hydrophilic PEO fragment of pluronic^®^ F-127 (F127) did not induce notable differences in its hemolytic activity. Conversely, the conjugation of Olp to the hydrophobic PPO fragments of mPEO_5k_-PPO_12k_ drastically decreased hemolytic activity of Olp (**Figure 1B**). According to the proton nuclear magnetic resonance (^1^H NMR) spectra in deuterium oxide (**Figure 1C**), the proton signals of Olp were visible for (mPEO-MA)_2_-Olp and F127-MA-Olp but invisible for (mPEO-PPO-MA)_2_-Olp. The decreased toxicity of (mPEO-PPO-MA)_2_-Olp was due to shielding of the Olp motifs. Hence, mPEO_5k_-PPO_12k_ nanoparticle could be served as a nanoblock for spatially shielding Olp and reducing its toxicity. (mPEO-PPO-MA)_2_-Olp was named ^NR^Nolp hereafter. ^NR^Nolp design also decreased the cytotoxicity of Olp, and ^NR^Nolp-treated 4T1 cells were both negative for Annexin V and PI staining, showing no obvious apoptosis or necrotic cell death (**Figure S4B**).

### Construction and characterization of ^R^Nolp

**Figure 2:**
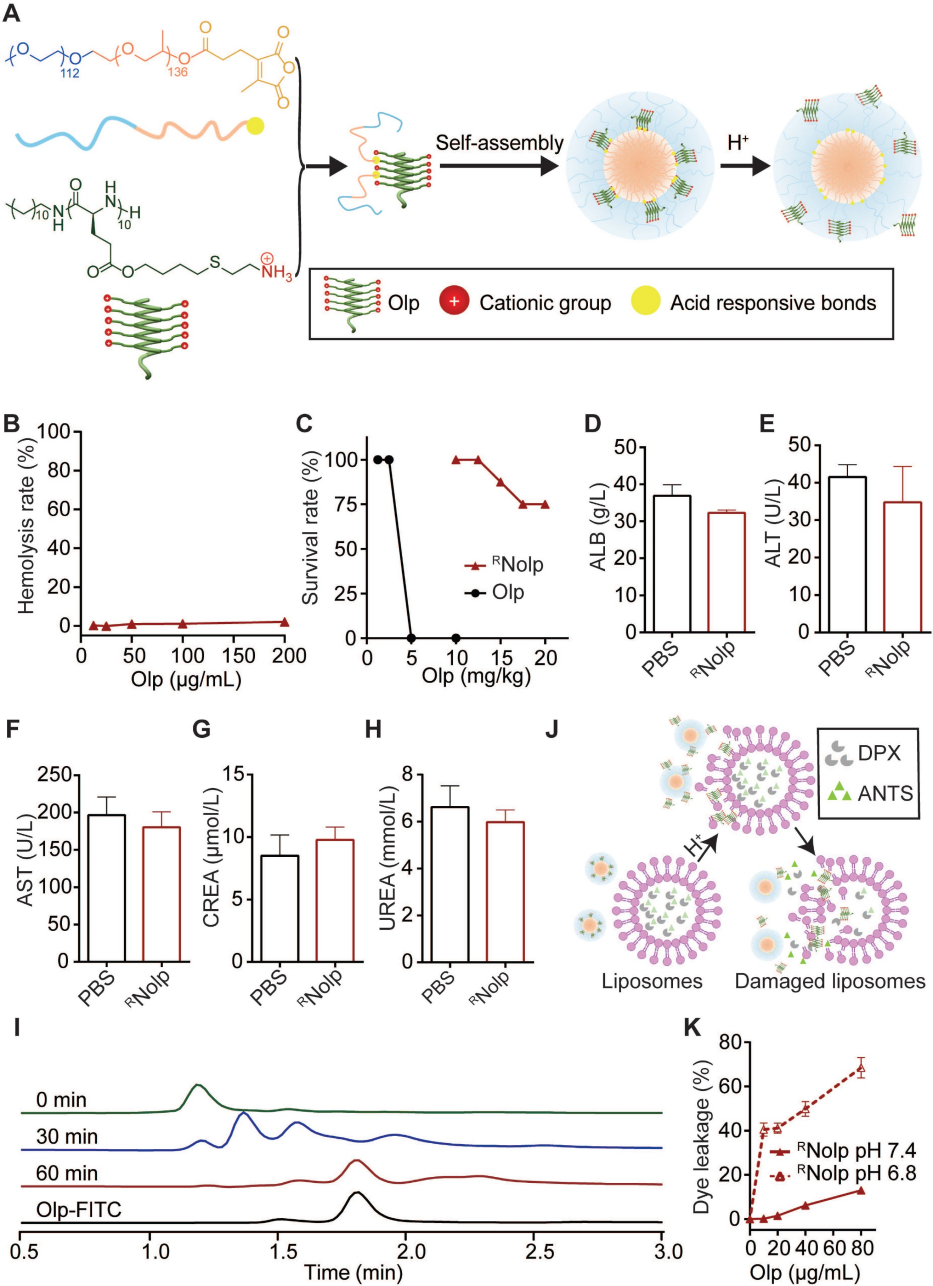
**The construction and characterization of ^R^Nolp. A**, Schematic illustration of the construction of tumor acidity-responsive ^R^Nolp and the acid-responsive release of Olp. **B**, Concentration-dependent hemolytic activity of ^R^Nolp at pH 7.4 after 1 h incubation. **C**, Survival rate of mice after i.v. injection with various doses of ^R^Nolp or Olp (8 mice per group) for 3 days. Concentration of albumin (ALB, **D**), alanine aminotransferase (ALT, **E**), aspartate aminotransferase (AST, **F**), serum creatinine (CREA, **G**), and UREA (**H**) in the serum of mice 7 days after i.v. injection with PBS or ^R^Nolp (6 mg/kg of Olp) daily for 3 days. **I**, Time-dependent release of Olp-FITC from ^R^Nolp after incubation in PBS buffer at pH 6.8. **J**, Schematic of acid-responsive liposome destruction and dye leakage by ^R^Nolp. **K**, Dye leakage measured of ANTS/DPX-encapsulated liposomes incubated with a series of concentrations of ^R^Nolp pretreated at pH 7.4 or pH 6.8. In **D**,** E**,** F**,** G** and **H**, data are shown as the mean ± s.e.m. (n = 4 independent mice). In **B** and **K**, data are presented as the mean ± s.d. (n = 3).

We next conjugated Olps to mPEO-PPO with acid-labile amide bonds (CDM) to obtain ^R^Nolp (**Figure 2A, Scheme S5 and Figure S11**), and ^NR^Nolp was used as the nonresponsive control. ^R^Nolp and ^NR^Nolp self-assembled into neutral nanoparticles of ~64 nm in diameter and the size of the nanoparticles remained stable in serum-containing phosphate-buffered saline (PBS) after 24 h of incubation (**Figure S12**). ^R^Nolp exhibited low hemolytic activity *in vitro,* similar to ^NR^Nolp (**Figure 2B**). The survival rate of mice intravenously injected with ^R^Nolp was substantially increased compared with that of Olp-injected mice (**Figure 2C**). The maximum tolerated dose without any treatment-related death increased by 5-fold in the ^R^Nolp-injected mice than that in the Olp-injected mice (**Figure 2C**). Moreover, 7 days following intravenous injection of PBS or ^R^Nolp (6 mg/kg Olp) daily for 3 days, mouse blood was collected for measuring serum ALT, AST, ALB, CREA, and UREA levels. Compared to the PBS-injected mice, no significant liver and kidney toxicity was detected for ^R^Nolp-injected mice (**Figure 2D-H**). The systemic toxicity of ^R^Nolp was further examined by H&E staining of the organs (heart, liver, spleen, lung, and kidney). No significant inflammatory response or pathological changes were observed in both PBS and treatment groups (**Figure S13**). After the incubation of ^R^Nolp at the characteristic tumor acidity of pH 6.8, the Olps were released over time (**Figure 2I and Figure S14**); while the particle size detected for the solution remained almost the same (**Figure S12A-C**). Correspondingly, ^R^Nolp exhibited tumor acidity-responsive membranolytic activity against cancer cell membrane-mimicking liposomes. ^R^Nolp induced extensive dye leakage at pH 6.8 but a low level of dye leakage at pH 7.4 (**Figure 2J-K**). As a control, the nonresponsive ^NR^Nolp did not release Olp at pH 6.8 (**Figure S15**) and induced low-liposome dye leakage at both pH 7.4 and pH 6.8 (**Figure S16**). Hence, the constructed ^R^Nolp exhibited decreased toxicity at physiological pH (pH 7.4) and selectively released Olps with membranolytic activity at tumor acidity (pH 6.8).

### ^R^Nolp exhibits tumor acidity responsive membranolytic activity and cytotoxicity against TNBC cells

**Figure 3:**
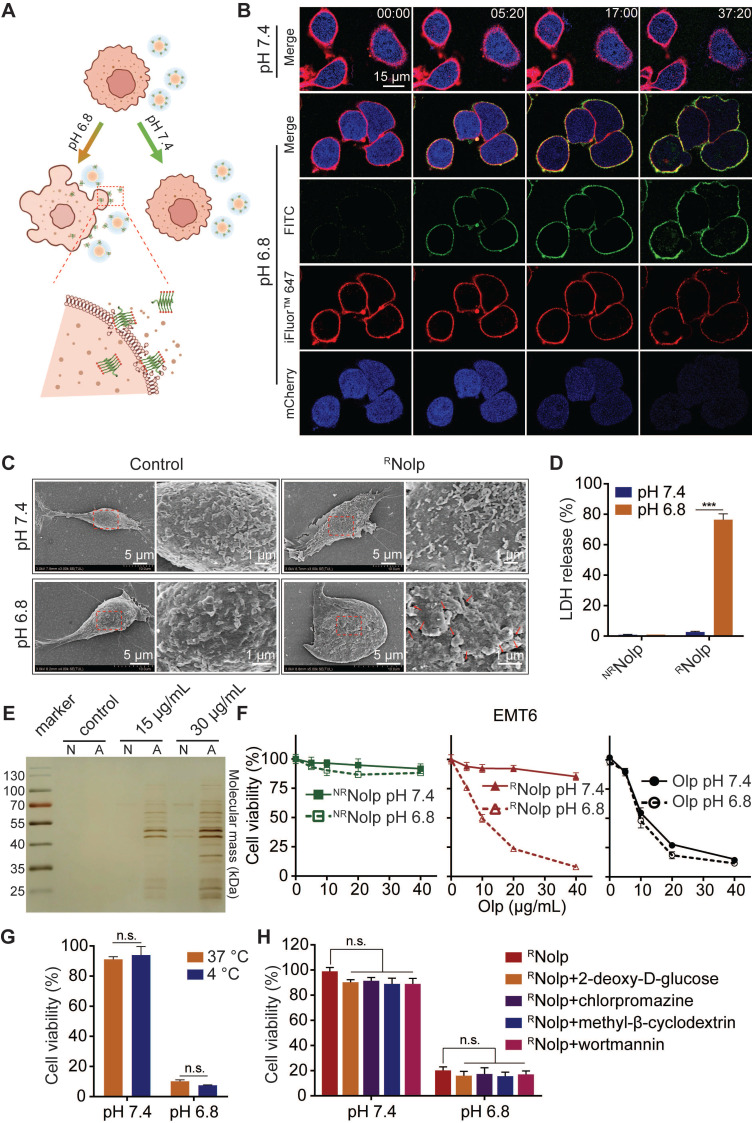
**^R^Nolp shows tumor acidity-responsive membranolytic activity and cytotoxicity against TNBC cells. A**, Schematic of the acid-responsive destruction of the plasma membrane and release of cell contents by ^R^Nolp. **B**, Confocal images of EMT6-mcherry cells, with mCherry (blue) expressed in the cytoplasm and cell membranes labeled with iFluor™ 647-WGA (red) treated with FITC-labeled ^R^Nolp (20 μg/mL of Olp) at pH 7.4 or pH 6.8 at different time points. **C**, SEM images of EMT6 cells treated with or without ^R^Nolp (20 μg/mL of Olp) at pH 7.4 or pH 6.8 for 30 min. The red arrows in the magnified image indicate holes in the cell membrane surface. **D**, LDH release of EMT6 cells treated with ^R^Nolp or ^NR^Nolp (40 μg/mL of Olp) at pH 7.4 or pH 6.8. **E**, Silver staining image of cell culture supernatants after incubation with ^R^Nolp (15 or 30 μg/mL of Olp) at pH 7.4 (N) or pH 6.8 (A) for 1 h. **F**, Acid-responsive cytotoxicity of ^R^Nolp. EMT6 cells were incubated with a series of concentrations of ^R^Nolp, ^NR^Nolp, or Olp at pH 7.4 or pH 6.8 for 1 h, and the cell viability was determined via the CCK-8 assay. **G**, Temperature-dependent cytotoxicity of ^R^Nolp. EMT6 cells were treated with ^R^Nolp (40 μg/mL of Olp) at pH 7.4 or pH 6.8 and incubated at 4 °C or 37 °C for 1 h. Then, the cell viability was determined via the CCK-8 assay. **H**, Cytotoxicity of ^R^Nolp against 4T1 cells at pH 7.4 or pH 6.8 after pretreatment with the endocytic inhibitors 2-deoxy-D-glucose (energy-dependent endocytosis inhibitor, 50 mM), chlorpromazine (clathrin-dependent endocytosis inhibitor, 10 μg/mL), methyl-β-cyclodextrin (caveolae-dependent endocytosis inhibitor, 50 μM), or wortmannin (macropinocytosis inhibitor, 50 nM). In **C**, representative SEM images are shown (at least three images were taken for each sample). In **B**, representative images from at least two independent experiments are shown. In **D**,** E**,** G** and **H** data are presented as the mean ± s.d. (n = 3); n.s. indicating no significant difference; *** P < 0.001.

^R^Nolp induced tumor acidity responsive plasma membrane rupture (PMR) of TNBC cells, releasing intracellular molecules and causing lytic cell death (**Figure 3A**). ^R^Nolp was incubated with TNBC cells at pH 7.4 or 6.8. At pH 7.4, ^R^Nolp-treated cells remained intact during the observation period (**Figure 3B**). At pH 6.8, Olp (FITC-labeled, green) attached to cell membranes (iFluor™ 647-WGA labeled, red). These cells swelled and developed bubble-like herniations that disintegrated abruptly, accompanied by the release of intracellular mCherry protein (blue) (**Figure 3B**). Scanning electron microscopy (SEM) observation revealed that ^R^Nolp treatment generated numerous holes in the cell membrane at pH 6.8 but not at pH 7.4 (**Figure 3C**). ^R^Nolp-mediated PMR at pH 6.8 released numerous intracellular proteins into supernatants, including large proteins, such as lactose dehydrogenase (140 kDa, a standard measure of PMR) (**Figure 3D-E**); treatment at pH 7.4 showed low levels of protein release (**Figure 3D-E**). Correspondingly, ^R^Nolp also showed tumor acidity-responsive cytotoxicity, with cytotoxicity at pH 6.8 comparable with that of Olp (**Figure 3F**). This cytotoxicity was independent of cell uptake and unaffected by incubation at 4 °C or endocytosis inhibitors (**Figure 3G-H**). ^R^Nolp thus demonstrates highly selective membranolytic activity and cytotoxicity against TNBC cells at the characteristic tumor acidity.

### ^R^Nolp inhibits tumor growth in orthotopic and metastatic mouse models of TNBC following intravenous administration

**Figure 4:**
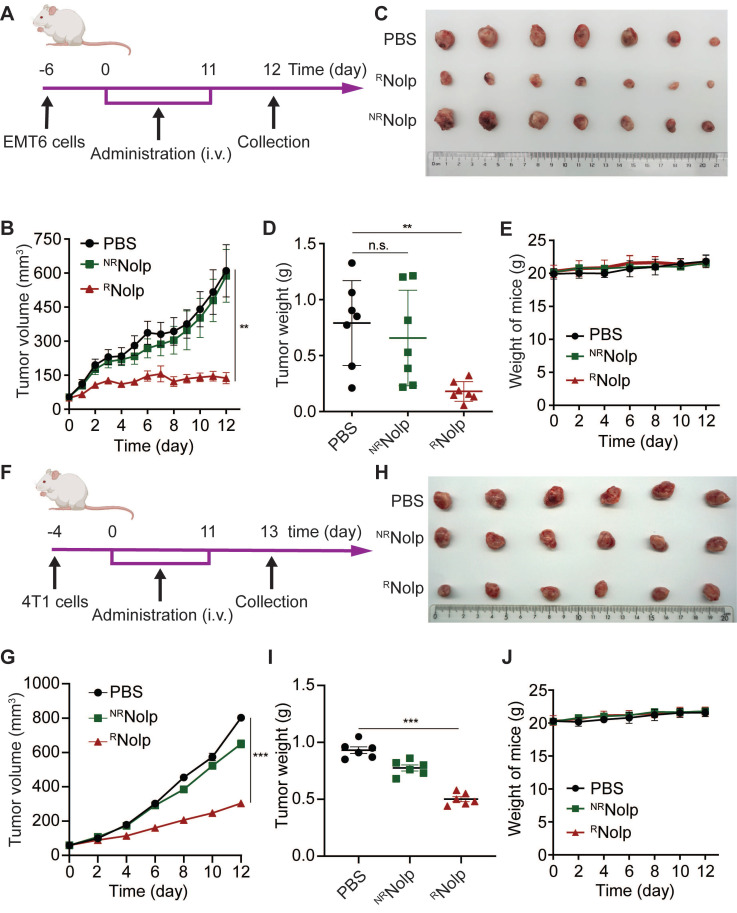
**^R^Nolp inhibits tumor growth in orthotopic mouse models of TNBC. A**, Treatment schedule for EMT6 tumors. BALB/c mice were orthotopically injected with EMT6 cells and i.v. injected with PBS, ^R^Nolp (6 mg/kg of Olp), or ^NR^Nolp (6 mg/kg of Olp) daily for 12 injections. On day 12 after the start of treatment, the tumors were collected. **B**, Tumor volume during treatment. Image (**C**) and weight (**D**) of collected tumors after treatment. **E**, Weight of mice during treatment. **F**, Treatment schedule for 4T1 tumors. BALB/c mice were orthotopically injected with 4T1 cells and i.v. injected with PBS, ^NR^Nolp (6 mg/kg of Olp) or ^R^Nolp (6 mg/kg of Olp) daily for 12 injections. On day 13 after the start of treatment, the tumors were collected. **G**, Tumor volume during treatment. Image (**H**) and weight (**I**) of collected tumors after treatment. **J**, Weight of mice during treatment. Data are presented as the mean ± s.e.m. (n = 6-7 independent mice); n.s. indicating no significant difference; ** P < 0.01, *** P < 0.001.

The anticancer efficacy of ^R^Nolp was evaluated in orthotopic and metastasis mouse models of TNBC. First, the orthotopic tumor model of EMT6 cells was established in the second left mammary fat pad of female BALB/c mice. These mice were then intravenously administered with either ^R^Nolp or ^NR^Nolp at an Olp dose of 6 mg/kg (**Figure 4A**). ^R^Nolp demonstrated a significant therapeutic effect on orthotopic EMT6 tumors (**Figure 4B-D**). The growth curves for tumor volume (**Figure 4B**) and the tumor weight (**Figure 4D**) at the end of the experiment were significantly decreased in ^R^Nolp-treated mice compared with tumors of the control and ^NR^Nolp-treated mice. Further, administering ^R^Nolp did not cause substantial weight loss in mice, suggesting that the mice tolerated the therapeutic dose well (**Figure 4E**). ^R^Nolp also showed a significant therapeutic effect on orthotopic 4T1 tumors and did not cause significant weight loss in the treated mice (**Figure 4F-J**). Then, the* in vivo* biodistributions of Cy5 labelled ^R^Nolp and ^NR^Nolp were studied in orthotopic mouse model of 4T1 tumor. The results showed that both ^R^Nolp and ^NR^Nolp were mainly distributed in liver, lung, kidney, spleen, and tumor at 1 h, 4 h and 24 h after intravenous injection. (**Figure S17**).

**Figure 5:**
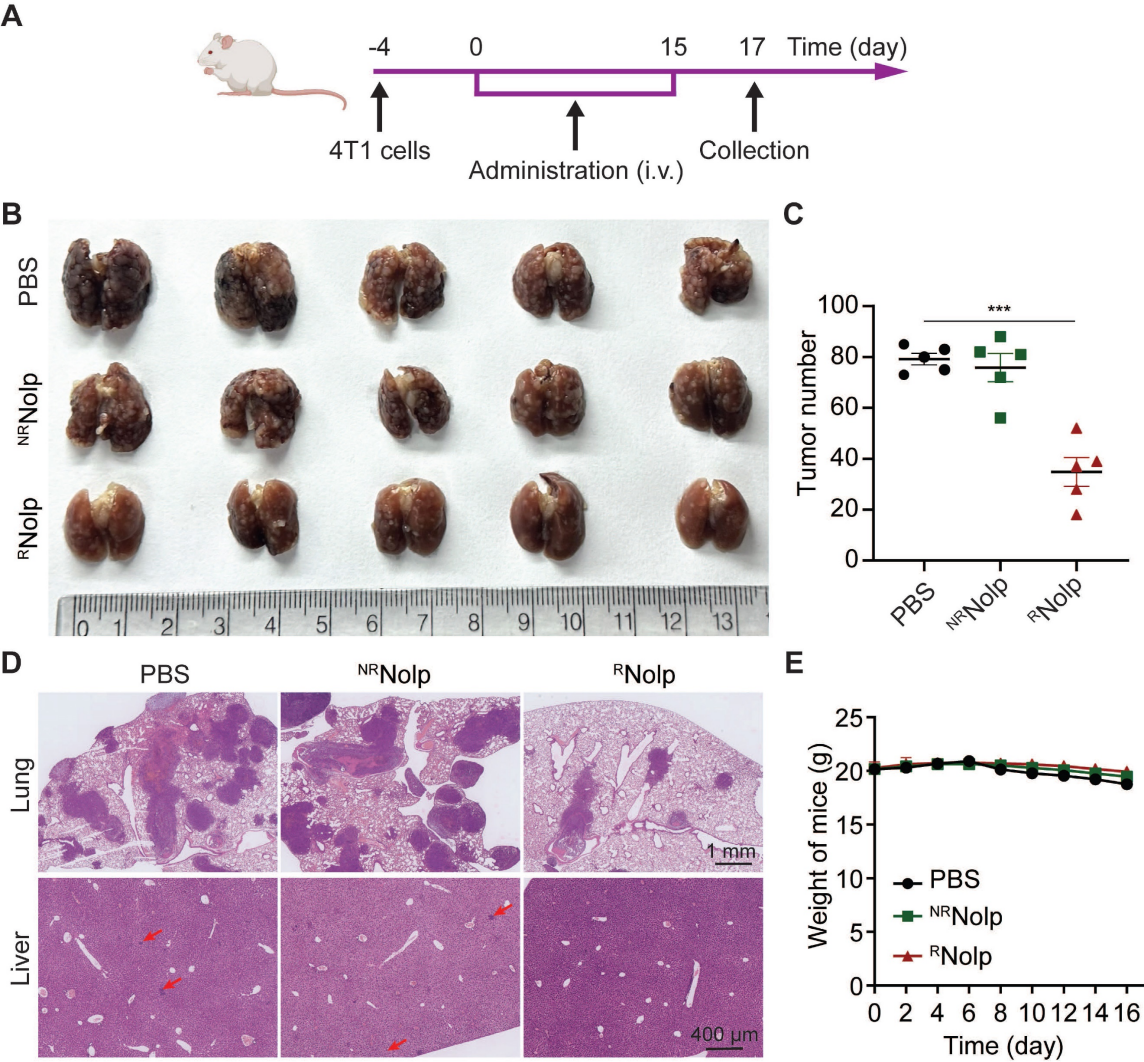
**^R^Nolp inhibits tumor growth in a metastasis mouse model of TNBC. A**, Treatment schedule for the 4T1 metastasis model. BALB/c mice were i.v. injected with 4T1 cells and i.v. injected with PBS, ^NR^Nolp (6 mg/kg of Olp) or ^R^Nolp (6 mg/kg of Olp) daily for 12 days and every other day for 4 days. On day 17 after treatment, the lung and liver were collected. Image (**B**) and surface nodule number of collected lung tissue (**C**) after treatment. **D**, H&E staining of lung and liver tissue after treatment. The scale bar is 1 mm and 400 μm for the section of lung and liver, respectively (Red arrows indicate liver metastases). **E**, Weight of mice during treatment. Data are presented as the mean ± s.e.m. (n = 5 independent mice); *** P < 0.001.

TNBC is characteristic of high metastasis to the tissues, such as lung and liver 35-37. Hence, we evaluated the therapeutic effect of ^R^Nolp in a metastatic mouse model. Mice were i.v. injected with 4T1 cells to establish a metastasis TNBC model, and i.v. administered with PBS, ^NR^Nolp or ^R^Nolp (**Figure 5A**). ^R^Nolp treatment significantly decreased the number of tumor nodules on the lung surface compared to the PBS and ^NR^Nolp groups (**Figure 5B-C**). H&E staining of the lung and liver also exhibited decreased metastasis nodule number and size in the ^R^Nolp-treated group compared to the control group (**Figure 5D**). And the administration of ^R^Nolp did not cause substantial weight loss in mice (**Figure 5E**).

## Conclusion

In conclusion, we developed a nanoblock-mediated strategy to achieve selective anticancer activity of Olps. In this system, Olps are shielded from interacting with cell membranes by a nanoblock with reduced toxicity in normal tissues and can be released to exert their potent membranolytic activity in the acidic tumor environment. This Olp shielded nanoblock exhibited high antitumor efficacy in orthotopic and metastatic mouse models of TNBC and showed low toxicity against normal tissues after intravenous administration. We provide an alternative strategy to increase the selectivity of Olps.

## Supplementary Material

Supplementary materials and methods, figures.

## Abbreviations

ALT: alanine aminotransferase
AST: aspartate aminotransferase
ALB: albumin
ANTS: 8-aminonaphthalene-1,3,6-trisulfonic acid
CDM: carboxy-dimethylmaleic anhydride
CREA: creatinine
DMEM: dulbecco's modified Eagle's medium
DPX: p-xylene-bis-pyridinium bromide
DMF: dimethylformamide
DOPE: 1,2-dioleoyl-sn-glycero-3-phosphoethanolamine
DMSO: dimethylsulfoxide
FITC: fluorescein-5-isothiocyanate
FBS: fetal bovine serum
^1^H NMR: proton nuclear magnetic resonance
ICR: institute of Cancer Research
LDH: lactate dehydrogenase
MBs: membranolytic blocks
MTT: thiazolyl blue tetrazolium bromide
mPEO-PPO: poly(ethylene oxide)-poly(propylene oxide)
^NR^Nolp: (mPEO-PPO-MA)_2_-Olp
^R^Nolp: (mPEO-PPO-CDM)_2_-Olp
Olps: oncolytic peptides
POPG: 1-palmitoyl-2-oleoyl-sn-glycero-3-phosphoglycerol
F127: pluronic^®^ F-127
PFA: paraformaldehyde
PBS: phosphate-buffered saline
PMR: plasma membrane rupture
RPMI: roswell park memorial institute
SEM: scanning electron microscopy
TNBC: triple-negative breast cancer
THF: tetrahydrofuran
iFluor™ 647-WGA: iFluor™ 647-Wheat Germ Agglutinin Conjugate
