# Supplementary material for: Nanoblock-mediated selective oncolytic polypeptide therapy for triple-negative breast cancer
Source: Theranostics. 2023 May 8;13(9):2800–10. doi: 10.7150/thno.81834 (PMC10240820; doi:10.7150/thno.81834)
Supplement: Supplementary file 1 — Supplementary materials and methods, figures. [file thnov13p2800s1.pdf]

Supporting Information

Nanoblock-mediated selective oncolytic polypeptide therapy for triple-negative breast cancer

Cuiyu Zhong[1,6#], Jie Li[5#], Suiping Liu[1,2,4], Weirong Li[1,2], Qiang Zhang[7], Junpeng Zhao[7], Menghua Xiong[5*], Yan Bao[2,4*], Yandan Yao[1,2,3*]

1. Breast Tumor Center, Sun Yat-sen Memorial Hospital, Sun Yat-sen University, Guangzhou 510120, China

2. Guangdong Provincial Key Laboratory of Malignant Tumor Epigenetics and Gene Regulation, Guangdong-Hong Kong Joint Laboratory for RNA Medicine, Medical Research Center, Sun Yat-Sen Memorial Hospital, Sun Yat-Sen University, Guangzhou 510120, China

3. Shenshan Medical Center, Sun Yat-sen Memorial Hospital, Sun Yat-sen University, Shanwei 516621, China

4. Nanhai Translational Innovation Center of Precision Immunology, Sun Yat-Sen Memorial Hospital, Foshan 528200, China

5. School of Biomedical Sciences and Engineering, South China University of Technology, Guangzhou International Campus, Guangzhou 511442, P. R. China

6. The Third Affiliated Hospital, Guangzhou Medical University, Guangzhou 510150, China

7. Faculty of Materials Science and Engineering, South China University of Technology, Guangzhou 510640, P. R. China

[#] These authors contribute equally.

[*]Corresponding authors: E-mail: xiongmh@scut.edu.cn (Menghua Xiong); baoy5@mail.sysu.edu.cn (Yan Bao); yaoyand@mail.sysu.edu.cn (Yandan Yao)

Additional Materials and Methods

Materials. L-Glutamic acid was purchased from Jill Biochemical Shanghai Co., Ltd. (Shanghai, China). 3-Butenyl alcohol, triphosgene, cysteamine hydrochloride, 8-aminonaphthalene-1,3,6-trisulfonic acid (ANTS), and p-xylene-bis-pyridinium bromide (DPX)

were purchased from Tokyo Chemical Industry Co., Ltd. (Shanghai, China). Dodecylamine, anhydrous dimethylformamide (DMF), 2,2-dimethoxy-2-phenylacetophenone, 1,2-dioleoyl-sn-glycero-3-phosphoethanolamine (DOPE), Sephadex G-50, and Triton X-100 were purchased from Sigma-Aldrich (Shanghai, China). 1-Palmitoyl-2-oleoyl-sn-glycero-3-phosphoglycerol (POPG) was purchased from CordenPharma (Liestal, Switzerland). Anhydrous dimethylsulfoxide (DMSO) was purchased from Sangon Biotech Co., Ltd. (Shanghai, China). Anhydrous ethyl acetate (EtOAc), tetrahydrofuran (THF), and hexane were dried by passing them through a solvent purification system (Vigor), which was connected to a glovebox (Vigor, Suzhou, China). Pluronic® F-127 was purchased from Sigma Life Science (Steinheim, Germany). Sulfo-Cyanine5 NHS ester was purchased from Confluore Biological Technology Co. (Xi'an, China). iFluor™ 647-Wheat Germ Agglutinin Conjugate (iFluor™ 647-WGA) was purchased from AAT Bioquest, Inc. (California, USA). Dialysis bags (MWCO = 14,000 Da) were purchased from Zhenjian Biotech Co., Ltd. (Shanghai, China). Microsep filters were purchased from Merck Millipore (Billerica, MA, USA). Fluorescein-5-isothiocyanate (FITC) was purchased from Aladdin. 1 M HEPES Buffer and Hank's Balanced Salt Mixture were purchased from Solarbio Technology Co., Ltd. (Beijing, China).

Fetal bovine serum (FBS), 0.25% trypsin, Roswell Park Memorial Institute (RPMI)-1640 medium, Dulbecco's modified Eagle's medium (DMEM), DMEM/F12 medium, and CCK-8 kits were purchased from Gibco (Grand Island, NY, USA). Penicillin-streptomycin, LDH cytotoxicity assay kit, and rapid silver stain kit were purchased from Beyotime Biotech Co., Ltd. (Shanghai, China). Annexin V-FITC/PI apoptosis detection kit was purchased from Elabscience Biotechnology Co., Ltd (Wuhan, China). Thiazolyl blue tetrazolium bromide (MTT) was purchased from Meilun Biotech Co., Ltd. (Dalian, China). Phosphate-buffered saline (PBS) was purchased from Boster Biological Technology Co., Ltd. (Wuhan, China). Fresh goat blood was purchased from Guangzhou Future Biotechnology Co., Ltd. (Guangzhou, China). PAGE gel rapid preparation kit was purchased from Epizyme Biotech Co., Ltd. (Shanghai, China). Wortmannin, 2-deoxy-D-glucose, chlorpromazine, and methyl-β-cyclodextrin were purchased from Macklin Biochemical Technology Co., Ltd. (Shanghai, China). Protein marker and cell culture flasks were purchased from Thermo Fisher Scientific (Bremen, Germany). Cell culture dishes (6 cm and 10 cm) and 96-well plates were purchased

from Corning Inc. (Corning, NY, USA). 4% paraformaldehyde (PFA) and 35 mm glass bottom dishes with a 15 mm micro-well were purchased from biosharp (Heifei, China).

Synthesis of mPEO-PPO-MA. mPEO-PPO (1.0 g, 0.057 mmol) was dissolved in dichloromethane and mixed with maleic anhydride (MA, 20 mg, 0.2 mmol) and 4-dimethylaminopyridine (DMAP) (10 mg, 0.08 mmol). Then, the solution was stirred for 12 h at room temperature. After concentration, the solution was precipitated in ether and centrifuged at low temperature to obtain mPEO-PPO-MA.

Characterization. The ^{1}H NMR spectra of the polymers were recorded on a Varian 400 MHz spectrometer. Chemical shifts were reported in ppm and referenced to the solvent proton impurities. Gel permeation chromatography (GPC) was used to characterize the polydispersity and molecular weight of C12-PButLG. The GPC instrument was equipped with an isocratic pump (Model 1515, Waters, Milford, MA, USA), refractive index detector (Model 2414, Waters), and size exclusion columns (HR0.5, HR1, HR2, and HR4 styrene divinylbenzene columns, 5 μm, 300 × 7.8 mm; Waters, Milford, MA, USA) at 30 °C using DMF as the mobile phase. The molecular weight and polydispersity were calculated using poly(ethylene glycol) as a standard sample. A CS30320 CD spectrometer (Applied Photophysics Ltd., England) was used to record the circular dichroism (CD) spectra. A Waters system equipped with a 2475 FLR detector and an analytical XBridge® C18 column (100 × 4.6 mm, Ireland) was used to perform high-performance liquid chromatography (HPLC). A Zetasizer system (Version 7.12, Malvern Panalytical Technologies Corporation, UK) was used to measure the size and zeta potential of nanoparticles via dynamic light scattering (DLS). A Spark™ Multimode microplate reader was used to measure hemolysis at 576 nm.

TEM observation of ^{R}Nolp and ^{NR}Nolp at pH 7.4 and 6.8. ^{R}Nolp and ^{NR}Nolp (6 mg/mL in DMSO) were added to deionized (DI) water dropwise while stirring (400 rpm). Then, the mixtures were dialyzed in DI water at room temperature for 4 h to remove DMSO (MWCO = 3500). The resultant mixtures were collected and diluted to the desired concentration. Using hydrochloric acid or sodium hydroxide, the pH values of the nanoparticle solutions were adjusted. ^{R}Nolp (0.5 mg/mL) or ^{NR}Nolp (0.5 mg/mL) at pH 7.4 and pH 6.8 were added dropwise to a copper mesh covered with carbon film, respectively. Then, the residual liquid

was removed with filter paper after 10 min. After drying at room temperature, 10 μL 3% uranyl acetate saturated solution (Henan Ruixin Experimental Supplies Co., Ltd.) was added dropwise for negative staining. After 2 min, the residual liquid was removed using filter paper. After drying at room temperature, TEM was performed with a JEOL JEM1400plus.

Characterization of the helicity of Olp. Olp, $(mPEO\text{-}MA)_2$-Olp, F127-MA-Olp, $(mPEO\text{-}PPO\text{-}MA)_2$-Olp, and $(mPEO\text{-}PPO\text{-}CDM)_2$-Olp were dissolved in DI water at a concentration of 0.1 mg/mL Olp. The solutions were then transferred to a quartz cuvette (path length = 0.05 mm) for CD tests.

Synthesis of Olp-FITC. C12-PButLG-CA (400 mg, 0.14 mmol) and triethylamine (15 mg, 0.14 mmol) were dissolved in methanol (10 mL). Then, FITC (55 mg, 0.14 mmol) was dissolved in 5 mL methanol and mixed with the C12-PButLG-CA solution at room temperature while stirring. After 24 h, the Olp-FITC was obtained via dialysis (MWCO = 1 kDa) against distilled water for 24 h followed by lyophilization (yield: 85%). And other fluorescent labeling materials can be prepared by replacing Olp with Olp-FITC.

Olp-FITC was released from ^{R}Nolp-FITC and ^{NR}Nolp-FITC. The ^{R}Nolp-FITC or ^{NR}Nolp-FITC were incubated with pH 6.8 buffer solution and analyzed by HPLC with a fluorescence detector at different time. The mobile phases were 50% methanol and 50% deionized water.

Stability of ^{R}Nolp and ^{NR}Nolp in FBS. ^{R}Nolp and ^{NR}Nolp nanoparticles were separately resuspended at 62.5 μg/mL in DMEM containing 10% FBS. Then, the nanoparticle size was measured at specific time points.

Preparation of ^{R}Nolp-Cy5 and ^{NR}Nolp-Cy5. ^{R}Nolp (62.5 mg/mL, corresponding to 5 mg/mL Olp in DMSO) and sulfo-cyanine5 NHS ester (1 mg/mL) were mixed in a molar ratio of Olp:Sulfo-Cyanine5 NHS ester = 10:4, and the mixture was incubated at room temperature for 1 h. Then, the mixture was added to PBS buffer (pH 8.0) dropwise under stirring (400 rpm). After stirring at 300 rpm for 1 h, the solution was dialyzed in PBS buffer (pH 7.4) at room temperature for 4 h and further purified with a Microsep (4 mL, 30 kDa). ^{NR}Nolp-Cy5 was prepared using the same method.

Cell culture. Cancer cell lines, namely EMT6, MDA-MB-231 and 4T1 were sourced from the

American Type Culture Collection (ATCC). The cells were cultured in RPMI-1640 or DMEM supplemented with 10% FBS, 100 U/mL penicillin, and 100 μg/mL streptomycin, and incubated at 37 °C and 5% CO_2 in a humidified atmosphere.

Hemolytic assay. Goat blood was centrifuged at 3000 rpm for 3 min to remove plasma. Then, it was washed 3 times with PBS buffer (pH = 7.4) and resuspended in PBS buffer (pH = 7.4). Erythrocyte suspensions were incubated with a series concentration of polymers for 1 h at 37 °C. Erythrocyte suspensions incubated with PBS and 0.2% Triton X-100 were used as negative and positive controls, respectively. After incubation, the erythrocyte suspensions were centrifuged at 3000 rpm for 3 min, and the supernatants were collected to measure the absorbance at 576 nm. Hemolysis rate was calculated as $[(\text{Absorbance}_{\text{sample}} - \text{Absorbance}_{\text{negative control}})/(\text{Absorbance}_{\text{positive control}} - \text{Absorbance}_{\text{negative control}})] \times 100\%$.

Liposome dye leakage assay. A mixture of lipids, DOPC (6 mg) and DPPS (1.93 mg) in a molar ratio of 3:1 was dissolved in chloroform (1 mL) and used to prepare the lipid film. The lipid film was hydrated with a dye solution (10 mM Na_2HPO_4, pH 7.0) containing ANTS (6.25 mM) and DPX (22.5 mM) using an ultrasound bath. This was followed by 10 successive freeze-thaw cycles in liquid nitrogen and warm water (37 °C). The resuspended lipid vesicles were then filtered through a polycarbonate membrane filter (400 nm) 20 times with a liposome extruder (Hamilton CO., Reno, Nevada), and purified through a Sephadex G-50 gel column (Beijing Separation Technology Co., Ltd., China). ^{NR}Nolp or ^{R}Nolp was pretreated for 1 h in pH 7.4 or pH 6.8 buffer (10 mM Na_2HPO_4, 90 mM NaCl) and diluted to a series of concentrations with the pH 7.4 buffer (10 mM Na_2HPO_4, 90 mM NaCl). Then, it was incubated with ANTS/DPX-encapsulated liposome in black 96-well plates for 15 min at 37 °C. Dye leakage was monitored using a microplate spectrophotometer (Tecan, Infinite M Plex, Switzerland). Liposome samples treated with 0.1% TritonX-100 were set as the positive control of 100% dye leakage. Those treated with buffer only (10 mM Na_2HPO_4, 90 mM NaCl) were set as the negative control. The samples without liposome but containing corresponding concentrations of ^{NR}Nolp or ^{R}Nolp were set as background controls. The formula for calculating dye release percentage is $[(I_{\text{sample}} - I_{\text{background control}} - I_{\text{negative control}})/(I_{\text{positive control}} - I_{\text{negative control}})] \times 100\%$.

Cytotoxicity assays. The CCK-8 assay was used to determine the cytotoxicity of ^{R}Nolp and ^{NR}Nolp. Tumor cells were seeded into 96-well plates at a density of 10^4 cells/well. After 24 h, the cell culture medium was replaced with fresh medium containing different concentrations of nanoparticles at pH 7.4 or pH 6.8. The wells treated without nanoparticles were set as the control of 100% survival. After 1 h incubation, the medium was replaced with fresh medium containing CCK-8 solution. After 3 h incubation, a microplate spectrophotometer was used to measure the absorbance of each well at 490 nm. The relative cell viability was calculated as $(A490_{sample} - A490_{blank})/(A490_{control} - A490_{blank}) \times 100\%$. $A490_{blank}$ was defined as the absorption of the medium.

To determine the cytotoxicity at different temperatures, the cells were treated with cell culture medium containing ^{R}Nolp (40 μg/mL equivalent concentration of Olp) at pH 7.4 or pH 6.8 and incubated at 4 °C or 37 °C for 1 h. Then, cell viability was determined via the CCK-8 assay.

For the cytotoxicity assays after endocytosis inhibition, the cells were pretreated with medium containing endocytic inhibitors, 2-deoxy-D-glucose (50 mM, Macklin, China), chlorpromazine (10 μg/mL, Macklin, China), methyl-β-cyclodextrin (50 μM, Macklin, China), or wortmannin (50 nM, Macklin, China) for 30 min at 37 °C. The cells were then treated with medium containing both ^{R}Nolp and endocytic inhibitors at pH 7.4 or pH 6.8 for 30 min. Cell viability was determined via the MTT assay (Catalog number MB4698, Meilune, China).

Transfection of mCherry into the EMT6 cell line. The mCherry expression plasmids were provided by Mingdong Liu, a postdoctoral researcher at the School of Biomedical Science and Engineering, South China University of Technology. The mCherry plasmids were packaged into lentiviruses by calcium phosphate-mediated co-transfecting with the virus packaging factors psPAX2 and pMD2.G into HEK293T cells. The recombinant lentivirus supernatant was collected after 48 hours. EMT6 cells were mixed with the lentivirus supernatant and puromycin (2 μg/mL) was added to screen the EMT6-mCherry cell line with mCherry expressed in the cytoplasm.

Confocal microscopy imaging. EMT6-mCherry cells were seeded on a confocal dish with a glass bottom 48 h before treatment. The cell culture medium was replaced with HHBS medium

containing 10 μg/mL iFluor™ 647-WGA and incubated for 8 min in the dark at room temperature. The HHBS medium (Hanks' Buffer with 20 mM HEPES) containing iFluor™ 647-WGA was removed and the cells were washed 3 times with fresh HHBS medium. Then, the DMEM medium at pH 7.4 or pH 6.8 was added to the confocal dish. ^{R}Nolp-FITC was subsequently added to the confocal dish with a final Olp concentration of 20 μg/mL. Images were captured every 20 s using the 40 × oil objective lens of an Olympus FV3000 confocal laser scanning microscope (Olympus Corporation, Tokyo, Japan). During observation, the confocal dishes were kept at 37 °C in a 5% CO_2 atmosphere.

Scanning electron microscopy (SEM) observation of ^{R}Nolp-treated cells. Before treatment, EMT6 cells were seeded onto a 12-well plate with 14-mm round glass coverslips at 10^5 cells/1 mL/well. Then, the cells were treated with cell culture medium containing ^{R}Nolp (20 μg/mL of Olp) at pH 7.4 or 6.8 and incubated at 37 °C for 30 min. The cells were washed 3 times with PBS and fixed with 2.5% glutaraldehyde, followed by dehydration in ethanol and drying in a freeze-drying device (JEOL-JFD-310, Japan). Finally, the cells on the coverslips were coated with gold and analyzed using SEM (Hitachi SU8010, Tokyo, Japan). Cells treated with the medium at pH 7.4 or 6.8 without ^{R}Nolp were used as negative controls.

LDH leakage assays. EMT6 cells were seeded and incubated onto a 96-well plate at 10^4 cells/100 μL/well for 24 h. The cell culture medium was replaced with fresh medium containing ^{R}Nolp or ^{NR}Nolp (40 μg/mL equivalent concentration of Olp) at pH 7.4 or pH 6.8. After 1 h incubation, the cell culture plate was centrifuged at 400 g for 5 min. The supernatants were collected to measure the LDH amount using an LDH assay kit according to the manufacturer's instructions. Untreated wells were set as a control for background LDH release, and the LDH-release reagent-treated wells were set as 100% LDH leakage. The equation to calculate the percentage of LDH release was $[(\text{Absorbance}_{\text{treated cells}} - \text{Absorbance}_{\text{background control}})/(\text{Absorbance}_{\text{LDH release reagent}} - \text{Absorbance}_{\text{untreated cells}})] \times 100\%$.

Silver staining of released content in the supernatant of ^{R}Nolp-treated cells. EMT6 cells were treated with different concentrations of ^{R}Nolp in FBS-free medium at pH 7.4 or 6.8 and incubated at 37 °C for 1 h. Cell culture supernatants were then collected and analyzed by sodium dodecyl sulfate-polyacrylamide gel electrophoresis (SDS-PAGE), followed by silver

staining using a silver staining kit (Beyotime, Shanghai), according to the manufacturer's instructions. Cancer cells treated without ^{R}Nolp were set as negative control.

***In vivo* biodistribution imaging.** 5×10^5 4T1 cells suspended in 100 μL PBS were injected orthotopically into the second left mammary fat pad of female BALB/c mice (6-7 weeks old). When the average tumor volume reached ~80 mm^3, the mice were i.v. injected with PBS (set as control group), ^{R}Nolp-Cy5 (6 mg/kg of Olp), or ^{NR}Nolp-Cy5 (6 mg/kg of Olp), respectively. After 1, 4, and 24 h, the mice were sacrificed and the tumor and main organs were harvested for *ex vivo* bioluminescent imaging. These tumors and organs were scanned using an IVIS Spectrum *in vivo* imaging system (PerkinElmer, Waltham, MA, USA) with an excitation bandpass filter at 620 nm and an emission at 670 nm. The results were analyzed by the Living Image software (Version 4.4, PerkinElmer, Waltham, MA, USA).

Evaluation of the toxicity of ^{R}Nolp and ^{NR}Nolp *in vivo*. Female ICR mice (6 weeks old) were randomly divided into 5 groups and i.v. injected with designed doses (3 mg/kg or 6 mg/kg equivalent concentration of Olp) of ^{R}Nolp, or ^{NR}Nolp daily for 5 days. The control group mice were i.v. injected with equal volume of PBS. 24 h after the last intravenous injection, mice were sacrificed and the hearts, livers, spleens, lungs and kidneys were harvested. These organs were fixed with 4% paraformaldehyde and then embedded in paraffin, which were subsequently cut into slices (4 μm) for the following H&E staining analysis (NanoZoomer S360, Hamamatsu Photonics, magnification: 400 ×).

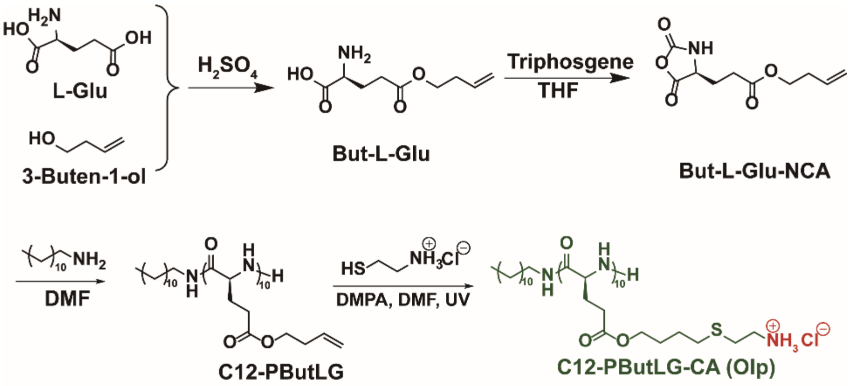

Scheme S1. The synthesis route of C12-PButLG-CA.

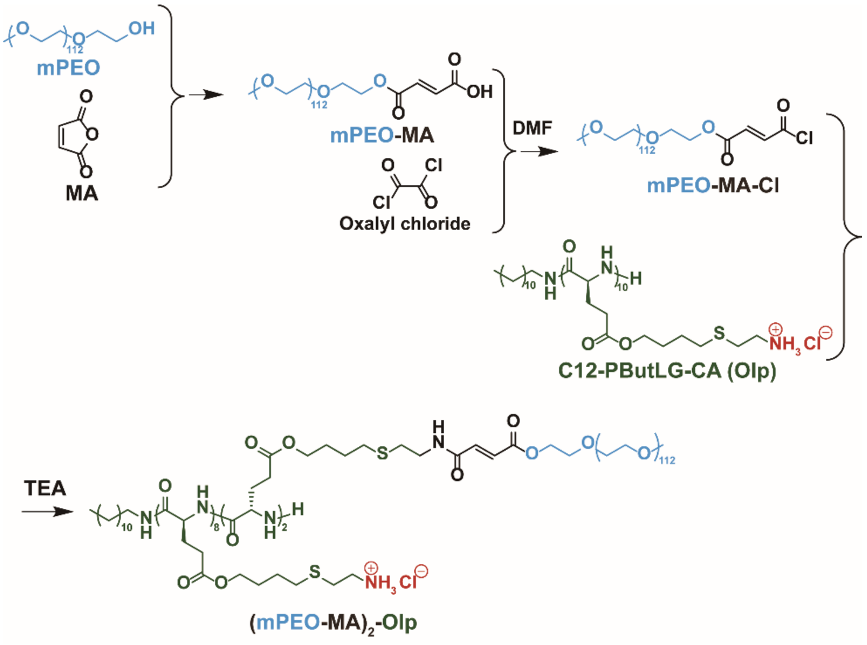

Scheme S2. The synthesis route of (mPEO-MA)$_2$-Olp

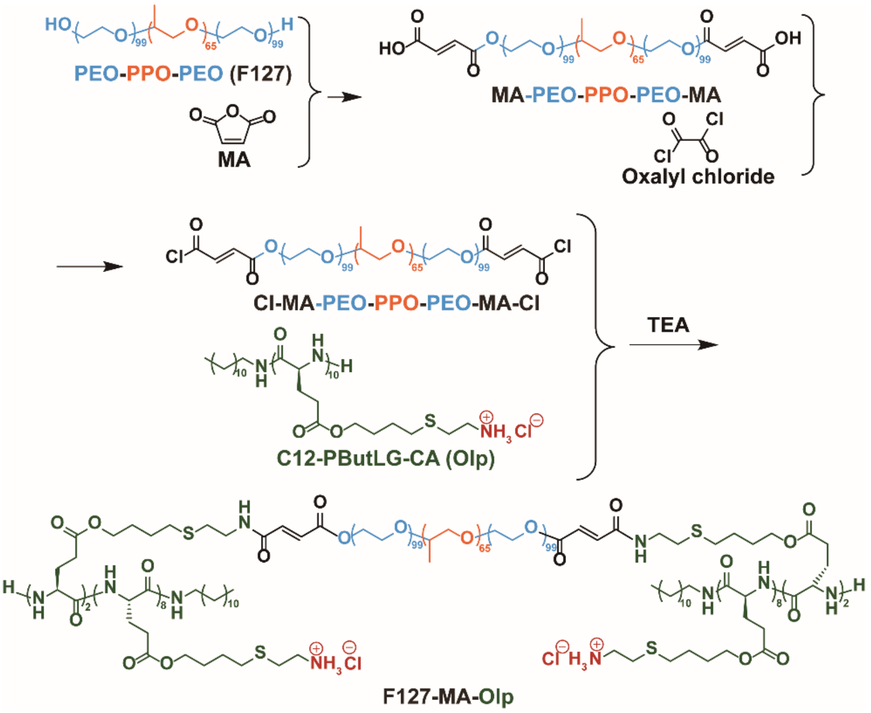

Scheme S3. The synthesis route of F127-MA-Olp

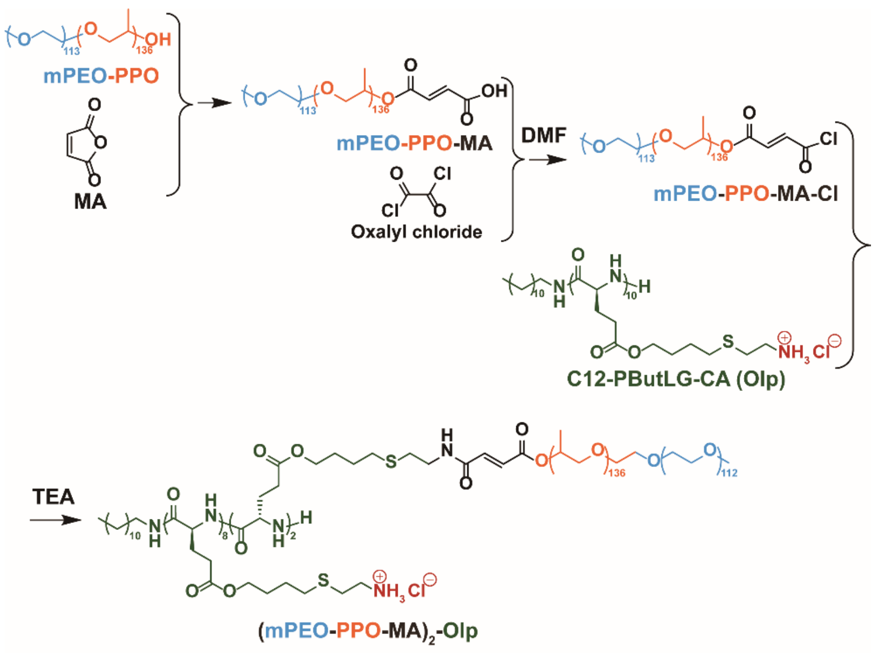

Scheme S4. The synthesis route of (mPEO-PPO-MA)2-Olp

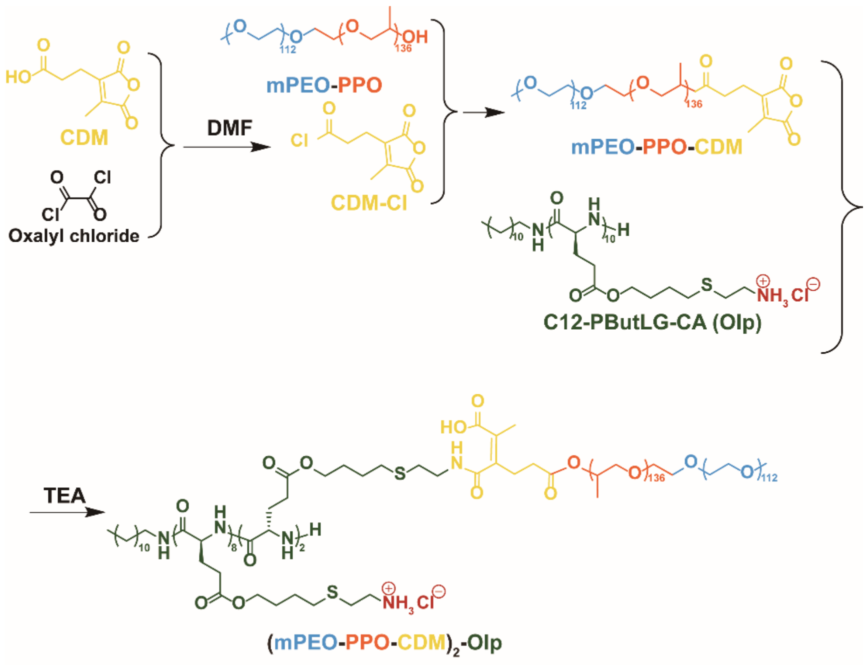

Scheme S5. The synthesis route of (mPEO-PPO-CDM)2-Olp

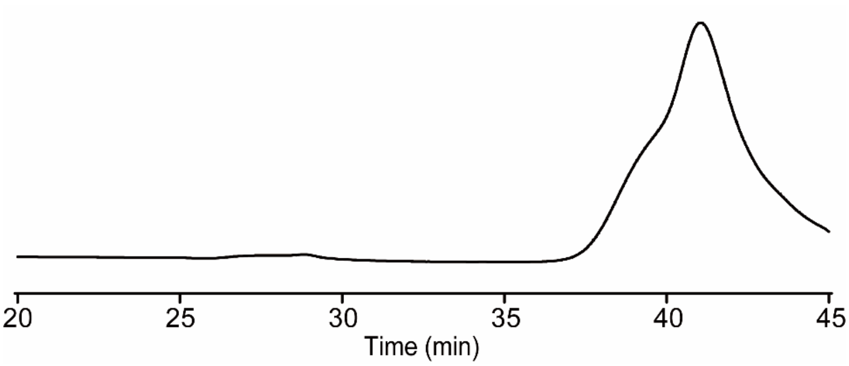

Figure S1. GPC curve of C12-PButLG

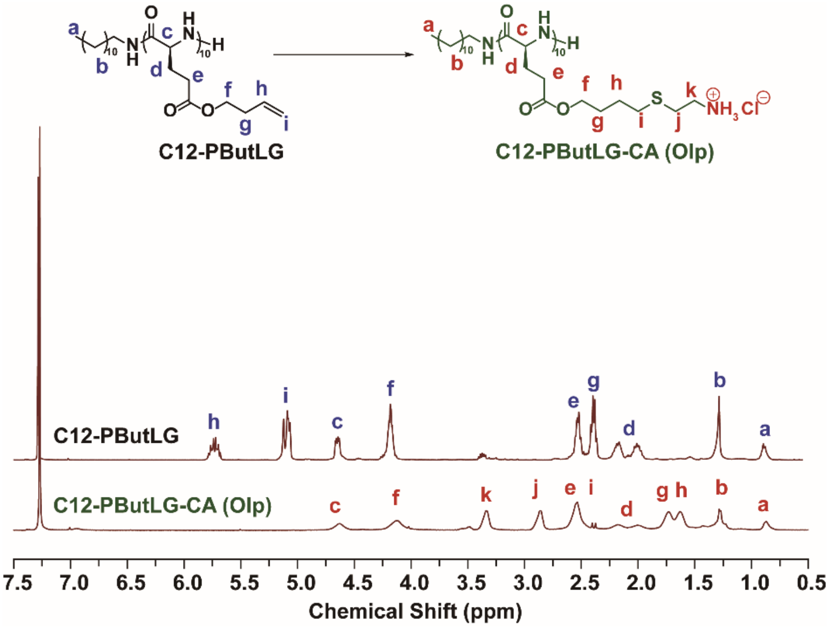

Figure S2. ^{1}H NMR spectra of C12-PButLG and C12-PButLG-CA in $CDCl_3$ and deuterated trifluoroacetic acid (TFA-d) (3:1) (ppm).

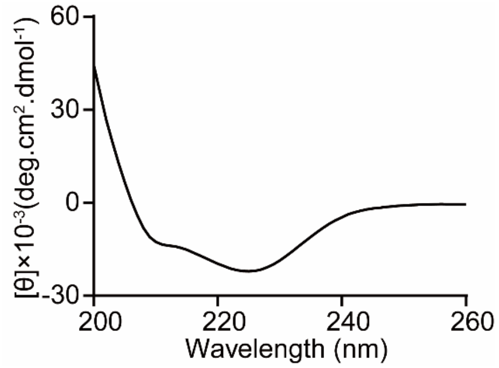

Figure S3. CD spectra of Olp

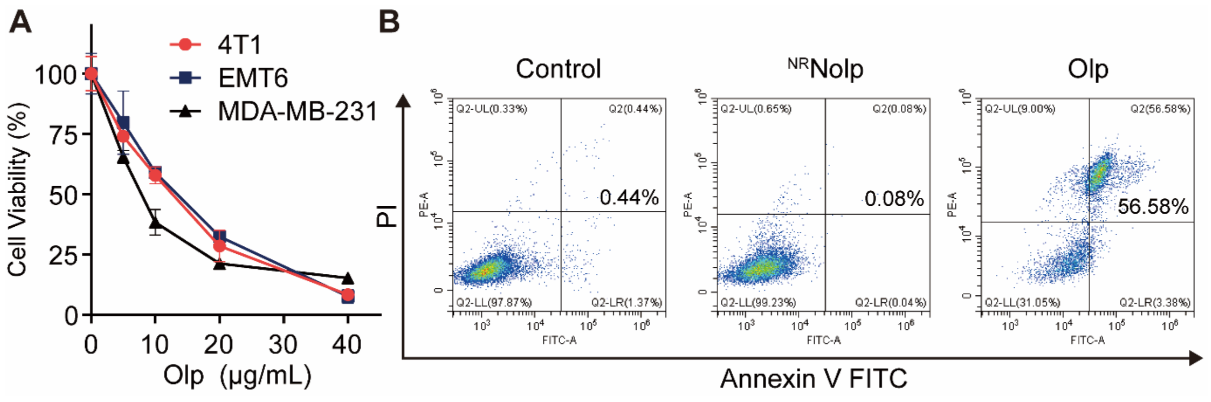

Figure S4. A, The concentration dependent cytotoxicity of the synthetic Olp against TNBC cells after 1 h of incubation. **B**, Annexin V FITC and PI staining of 4T1 cell after 24 h of treatment with Olp (5 μg/mL) or ^{NR}Nolp (5 μg/mL of Olp).

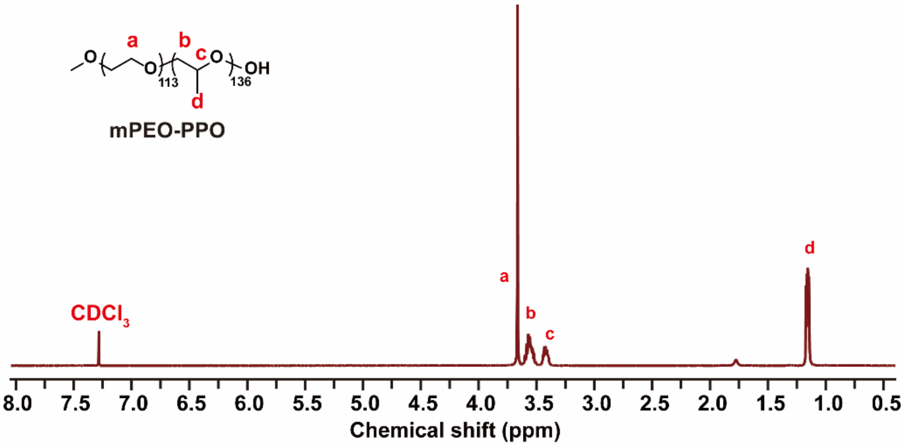

Figure S5. ^{1}H NMR spectrum of mPEO-PPO in $CDCl_3$ (ppm).

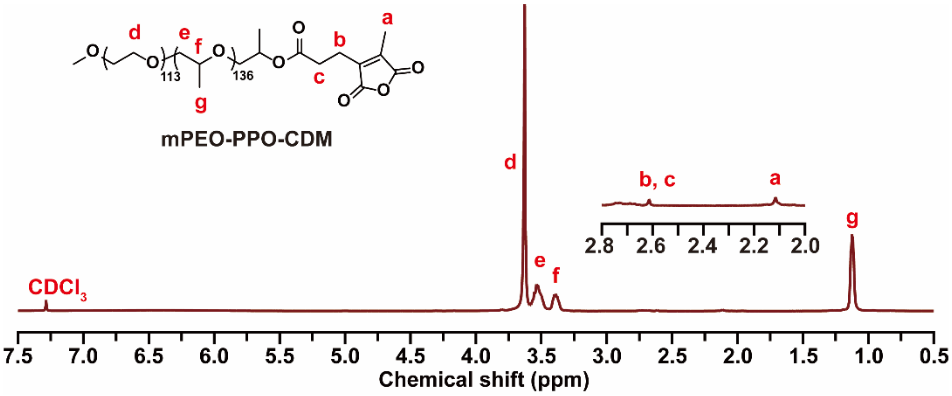

Figure S6. ^{1}H NMR spectrum of mPEO-PPO-CDM in $CDCl_3$ (ppm).

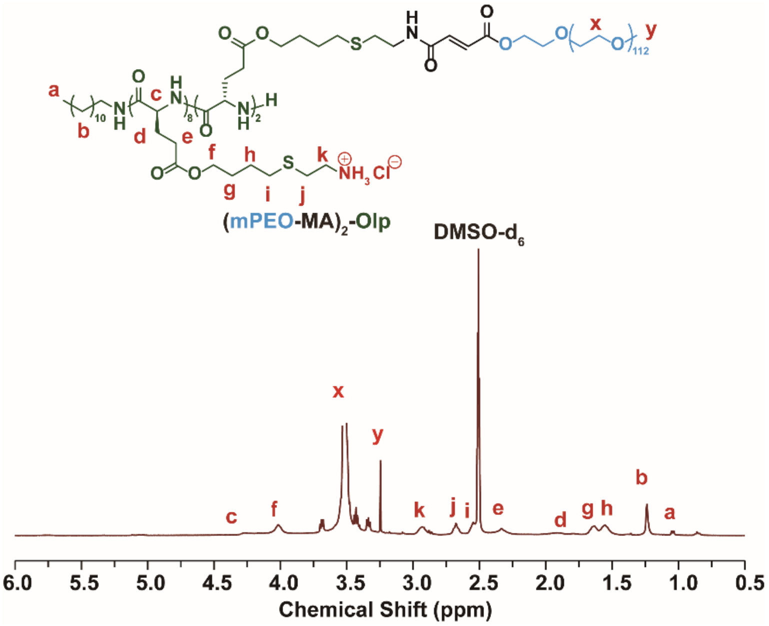

Figure S7. ^{1}H NMR spectrum of (mPEO-MA)$_2$-Olp in DMSO-d$_6$ (ppm).

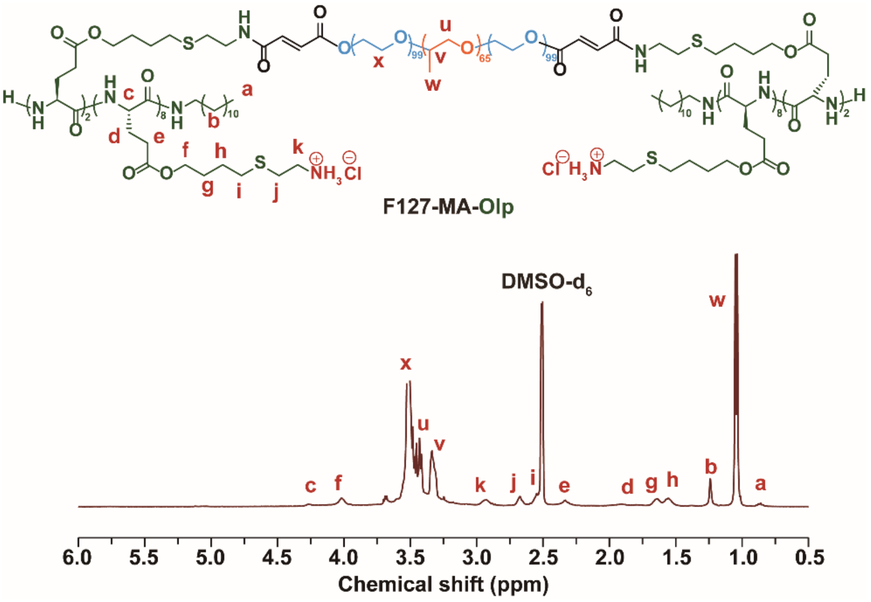

Figure S8. ^{1}H NMR spectrum of F127-MA-Olp in DMSO-d$_6$ (ppm).

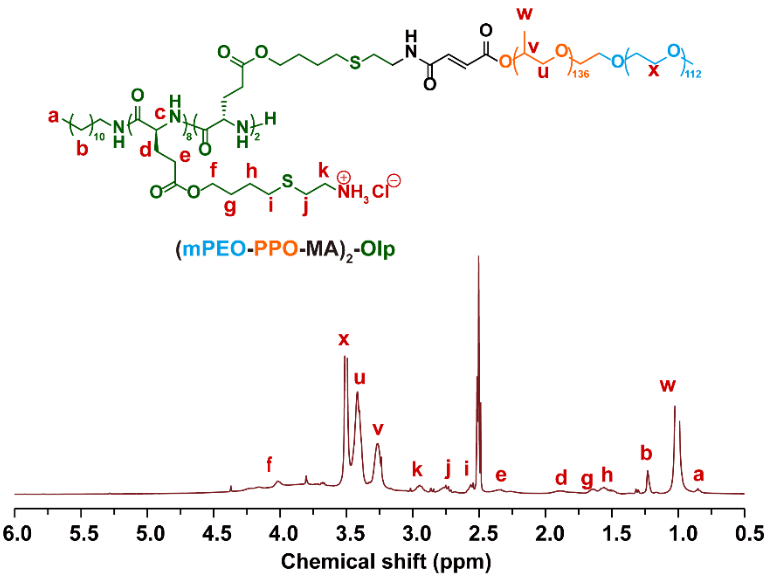

Figure S9. ^{1}H NMR spectrum of $(mPEO\text{-}PPO\text{-}MA)_2$-Olp in DMSO-$d_6$ (ppm).

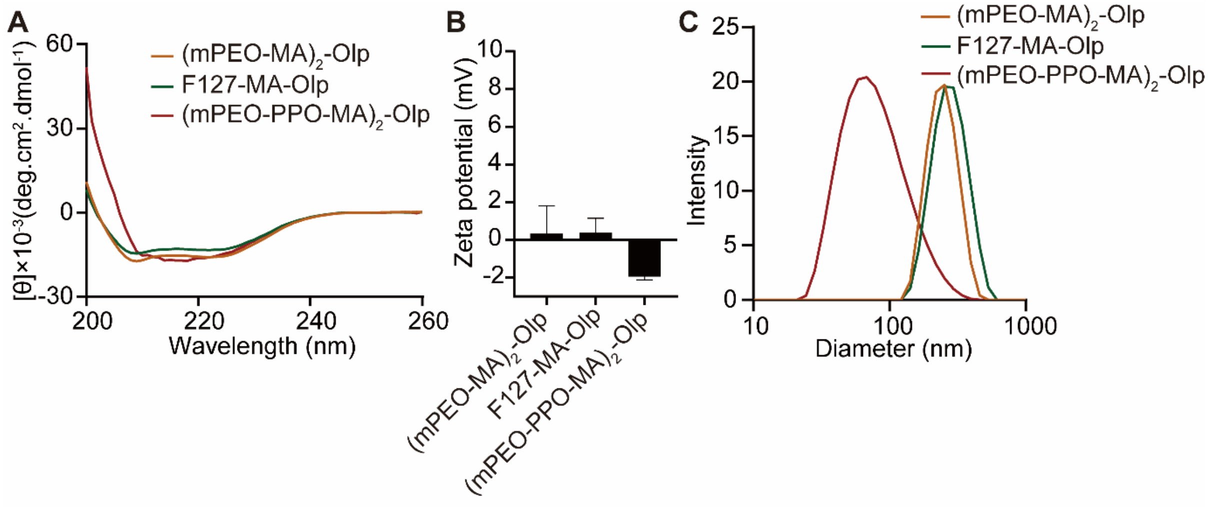

Figure S10. The CD spectra (**A**), zeta potential (**B**) and size (**C**) of $(mPEO\text{-}MA)_2$-Olp, F127-MA-Olp, and $(mPEO\text{-}PPO\text{-}MA)_2$-Olp.

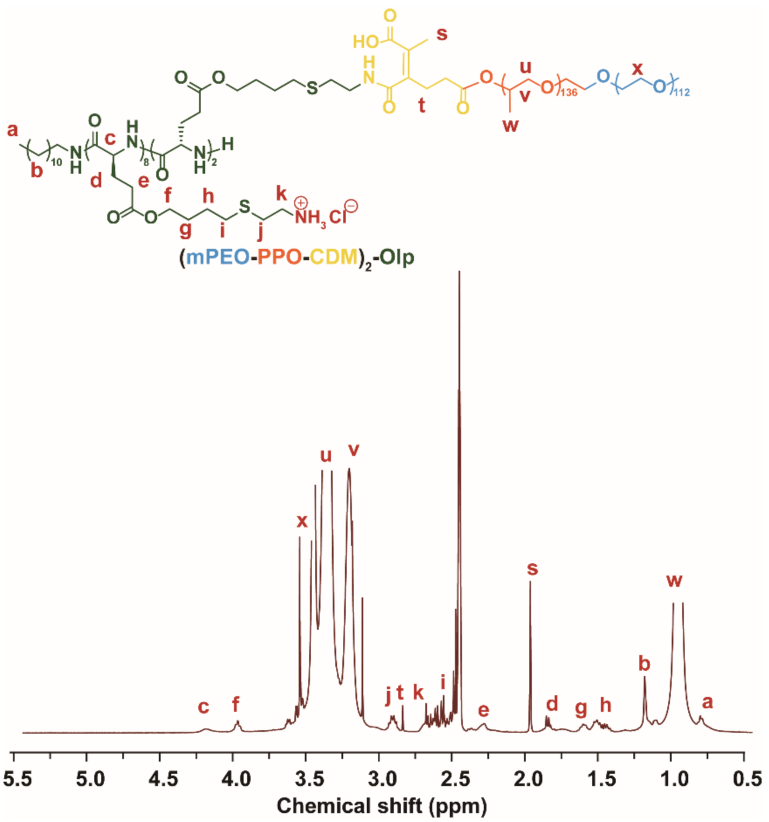

Figure S11. ^{1}H NMR spectrum of (mPEO-PPO-CDM)2-Olp in DMSO-d$_6$ (ppm).

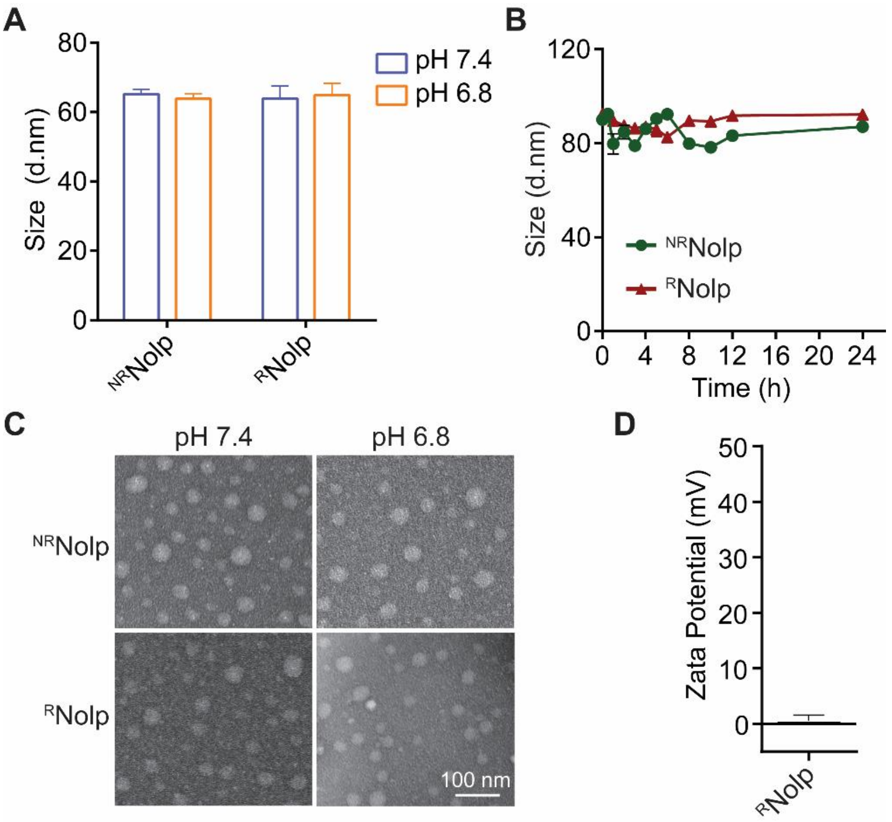

Figure S12. A, The size of ^{R}Nolp and ^{NR}Nolp in PBS buffer at pH 7.4 and pH 6.8. **B**, Changes in size of ^{R}Nolp and ^{NR}Nolp over time in medium containing 10% FBS. **C**, Images of ^{R}Nolp

and ^{NR}Nolp at pH 7.4 and 6.8 by TEM. Scale bars, 100 nm. **D**, Zeta potential of ^{R}Nolp.

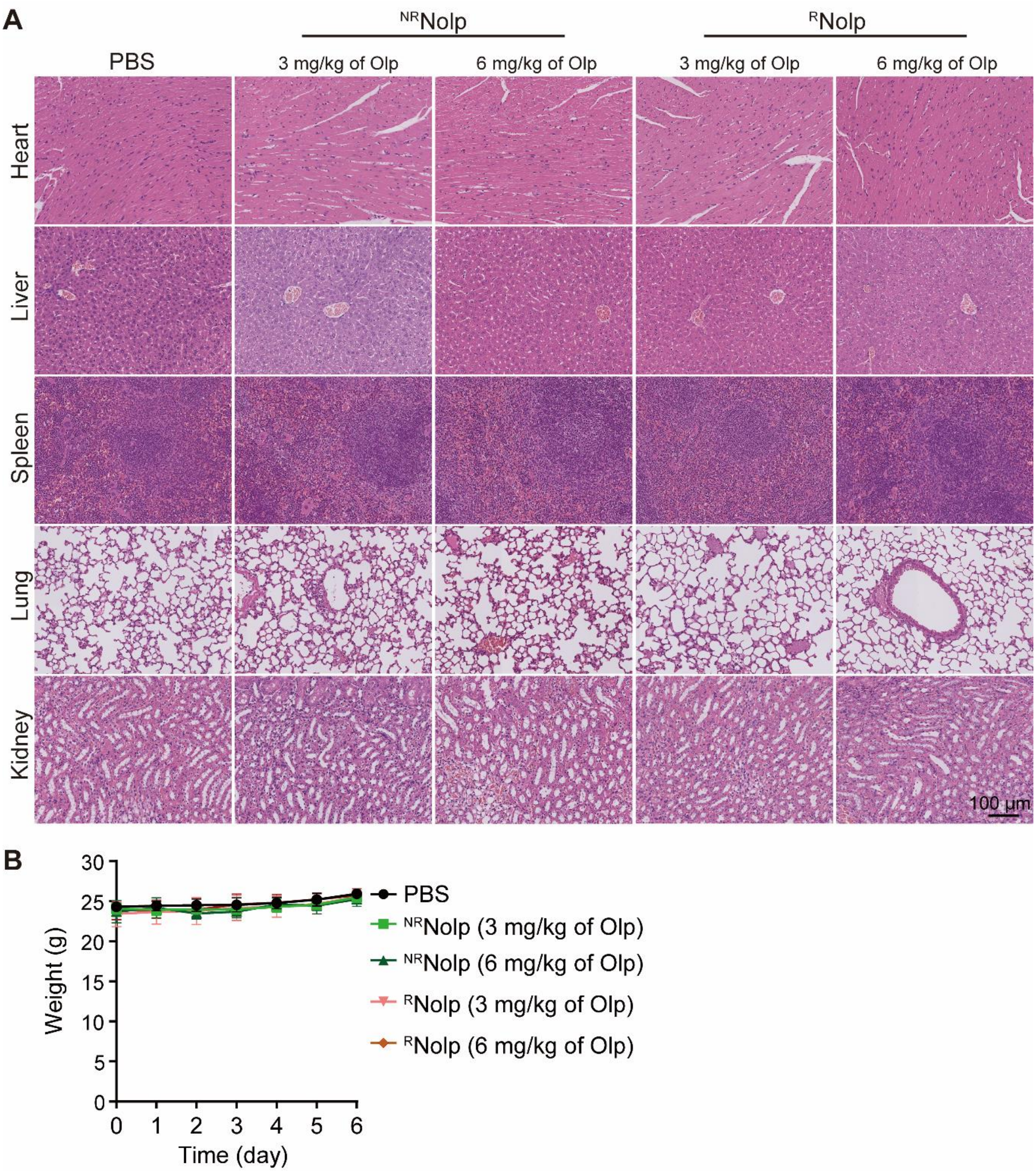

Figure S13. A, H&E staining of main organs (heart, liver, spleen, lung and kidney) of the ICR mice treated with different concentrations of ^{R}Nolp or ^{NR}Nolp (magnification × 400, scale bar: 100 μm). **B**, Weight of mice during treatment. All of the above concentrations represent the concentration of Olp contained in the ^{R}Nolp or ^{NR}Nolp. Data are presented as the mean ± s.e.m. (n = 3 independent mice).

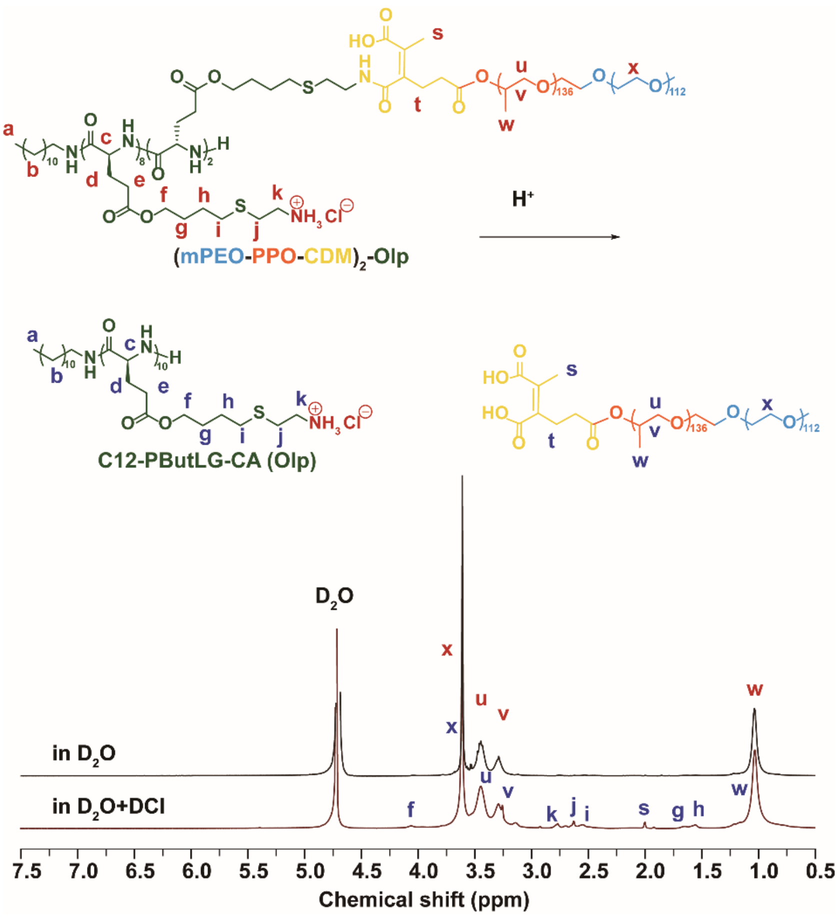

Figure S14. ^{1}H NMR spectra of (mPEO-PPO-CDM)$_2$-Olp in D_2O and D_2O with DCl (ppm).

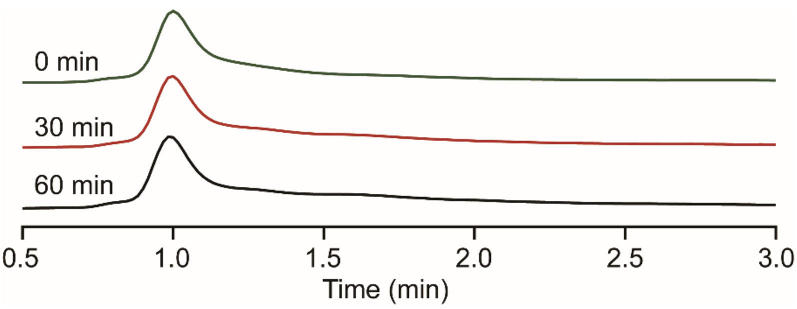

Figure S15 Time-dependent release of Olp-FITC from ^{NR}Nolp after incubation in PBS buffer at pH 6.8.

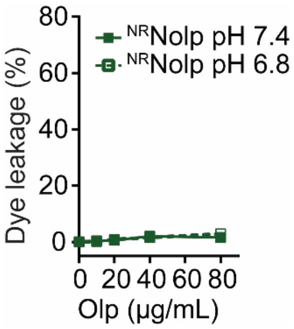

Figure S16. Dye leakage measured of ANTS/DPX-encapsulated liposomes incubated with a series of concentrations of ^{NR}Nolp pretreated at pH 7.4 or pH 6.8.

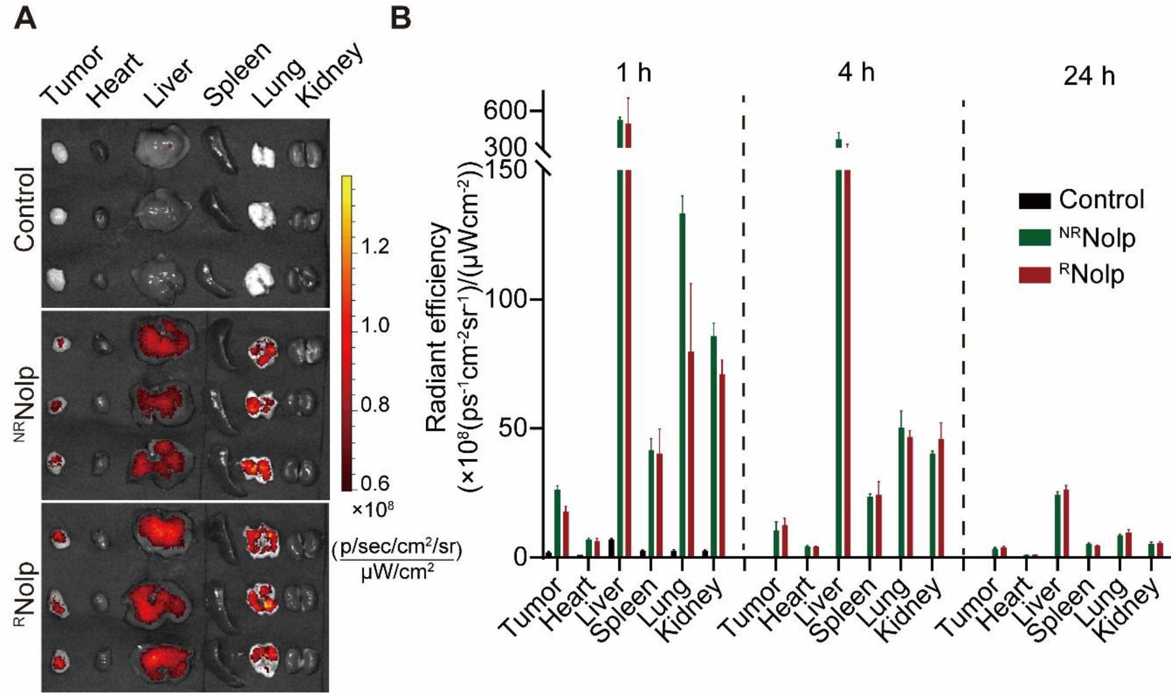

Figure S17. The *in vivo* biodistribution of Cy5 labeled ^{R}Nolp and ^{NR}Nolp in orthotopic mouse model of 4T1 tumor. The tumor, heart, liver, spleen, lung and kidney were collected at 1, 4, and 24 h after intravenous injection of ^{R}Nolp-Cy5 and ^{NR}Nolp-Cy5 at a dose of 6 mg/kg of Olp and imaged by the IVIS Spectrum *in vivo* imaging system. **A**, The average fluorescence intensity of major organs and tumors. **B**, The *ex vivo* images of tumor and major organs at 24 h after injection.
